# Peptide Release after Simulated Infant In Vitro Digestion of Dry Heated Cow’s Milk Protein and Transport of Potentially Immunoreactive Peptides across the Caco-2 Cell Monolayer

**DOI:** 10.3390/nu12082483

**Published:** 2020-08-18

**Authors:** Hannah E. Zenker, Harry J. Wichers, Monic M. M. Tomassen, Sjef Boeren, Nicolette W. De Jong, Kasper A. Hettinga

**Affiliations:** 1 Food Quality & Design Group, Wageningen University & Research Centre, 6708 WG Wageningen, The Netherlands; hannah.zenker@wur.nl; 2 Wageningen Food & Biobased Research, Wageningen University & Research Centre, 6708 WG Wageningen, The Netherlands; harry.wichers@wur.nl (H.J.W.); monic.tomassen@wur.nl (M.M.M.T.); 3 Laboratory of Food chemistry, Wageningen University & Research Centre, 6708 WG Wageningen, The Netherlands; 4 Laboratory of Biochemistry, Wageningen University & Research Centre, 6708 WE Wageningen, The Netherlands; sjef.boeren@wur.nl; 5 Internal Medicine, Department of Allergology & Clinical Immunology, Erasmus Medical Centre, 3000 CA Rotterdam, The Netherlands; n.w.dejong@erasmusmc.nl

**Keywords:** cow’s milk protein, peptides, Caco-2 cell, immunogenicity, allergenicity, glycation

## Abstract

Dry heating of cow’s milk protein, as applied in the production of “baked milk”, facilitates the resolution of cow’s milk allergy symptoms upon digestion. The heating and glycation-induced changes of the protein structure can affect both digestibility and immunoreactivity. The immunological consequences may be due to changes in the peptide profile of the digested dry heated milk protein. Therefore, cow’s milk protein powder was heated at low temperature (60 °C) and high temperature (130 °C) and applied to simulated infant in vitro digestion. Digestion-derived peptides after 10 min and 60 min in the intestinal phase were measured using LC-MS/MS. Moreover, digests after 10 min intestinal digestion were applied to a Caco-2 cell monolayer. T-cell epitopes were analysed using prediction software, while specific immunoglobin E (sIgE) binding epitopes were identified based on the existing literature. The largest number of sIgE binding epitopes was found in unheated samples, while T-cell epitopes were equally represented in all samples. Transport of glycated peptide indicated a preference for glucosyl lysine and lactosyl-lysine-modified peptides, while transport of peptides containing epitope structures was limited. This showed that the release of immunoreactive peptides can be affected by the applied heating conditions; however, availability of peptides containing epitopes might be limited.

## 1. Introduction

Cow’s milk protein consists of two major protein fractions, casein and whey protein, and is an important protein source in infant nutrition. Heating and glycation of cow’s milk protein (MP) has been shown to alter its digestibility and immunogenicity. Dry heating, as applied in this study, is not commonly used in the dairy industry; however, it has an important role in mimicking the heat treatment when MP powder is baked into a muffin or waffle. These products are often referred to as “baked milk” and have been shown to accelerate the resolution of cow’s milk allergy symptoms in allergic children [1]. Under these heating conditions (low aw-level, high sugar content, high temperature), protein aggregation and modifications via the Maillard reaction (MR) are favored [2]. The MR is the reaction between primary amino-groups of proteins, peptides and amino acids and the reactive carbonyl group of reducing sugars, for instance lactose. During the early stage of the MR, the initial condensation to the Amadori product occurs followed by a rearrangement to lactosyl lysine or glucosyl lysine. In the advanced stage of the MR, a pool of different advanced glycation end products (AGEs) are formed [3]. Amongst these, N^ε^-carboxymethyllysine (CML) has been used as a marker for the advanced stage of the MR and is one of the most abundant AGEs in processed dairy products [4,5]. The extent of the MR can affect the digestibility and immunoreactivity of MP. With respect to digestibility, the effect of thermal processing of milk and dairy products on peptide generation during gastrointestinal digestion in vitro and in vivo has been subject to several studies [6,7,8,9,10,11]. Moreover, it was shown that lysine blockage via the MR affects peptide size distribution after simulated infant in vitro digestion of infant formula [12] and that glycation of isolated milk proteins changes the composition of the peptides in digestion [13]. Heating and glycation can also affect immunogenicity and allergenicity of MP [14]. For example, binding of specific immunoglobulin E (sIgE) to either isolated MP or MP in mixture has been shown to decrease for extensively glycated milk proteins, possibly related to a masking effect on epitopes [15,16,17]. However, the effects of glycation and protein aggregation under the applied heating conditions are difficult to disentangle and can both affect sIgE binding [18]. Corzo-Martínez et al. [19] also showed that impaired digestibility may increase the residual allergenicity after in vitro digestion, when comparing heat-glycated and unheated β-lactoglobulin. Differences in the peptide profiles after ingestion of dry heated MP vs. unheated MP could affect the immunological response by differential preservation or glycation induced-modification of linear sIgE binding epitopes. Moreover, the generation of peptides carrying a glycation structure can modulate the inflammatory response by binding to the receptors for AGEs on antigen presenting cells [14,20]. Binding of AGEs to AGE receptors has particularly been shown for protein-bound CML and pyrroline [21,22], while for peptide bound AGEs this was only demonstrated for CML [20]. The availability of AGE-modified peptides to the gastrointestinal immune system by means of translocation across the epithelial barrier is an important determinant in the immunological response to a foreign antigen. The metabolic transit of AGEs has been shown in previous literature on the excretion of CML and pyrroline in urine [23,24]. Moreover, translocation across the Caco-2 cell monolayer has been shown for lactosylated and CML-modified dipeptides [25]. As reviewed by O’Hagan et al. [26], the literature reports that small quantities of intact proteins, other macromolecules, and intact antigens can pass the intestinal epithelial layer in vivo. Furthermore, the identification of cow’s milk derived peptides, ranging from 6 to 17 amino acids, in human milk has recently been described, indicating their absorption via the gastrointestinal tract [27]. However, to our knowledge the transport of food derived glycated peptides larger than two amino acids has not yet been investigated. Transport of larger glycated peptides can be an important factor for the binding of AGE-modified peptides as it has been suggested that CML is more abundant in fractions of in vitro digests that are larger than 1 kDa [28]. Therefore, transport of larger AGE-modified peptides could also contribute to the pool of dietary derived AGEs. This could be crucial as it has been shown with the example of CML that binding to AGE receptors is dependent on the concentration in which the CML is present in the vicinity of the receptors [22]. In this study, the peptide profiles of low temperature (LT) and high temperature (HT) heated MP after simulated infant in vitro digestion was compared to that of non-treated milk (NT). The most abundant glycation induced post translational modifications (PTMs), including CML and pyrroline as potential AGE receptor ligands, were monitored before and after transport across a Caco-2 cell monolayer. Special attention was given to sIgE binding epitopes and T-cell epitopes to assess immunomodulatory potential of the digest on the peptide level.

## 2. Materials and Methods

### 2.1. Chemicals

Dulbecco’s Modified Eagles Medium supplemented with high glucose, HEPES, l-glutamine (42,430,082), both with and without phenol red as well as trypsin-EDTA (0.25%) with phenol red, and HyClone™ Fetal Bovine Serum were purchased from Thermo Fisher Scientific (Waltham, MA, USA). All other chemicals were obtained from Merck KGaA (Darmstadt, Germany).

### 2.2. Preparation of Milk Powders and Heat Treatment

Liquid raw cow’s MP concentrate was obtained from FrieslandCampina (Wageningen, The Netherlands) and was composed of micellar casein (MCI88 liquid) and whey protein (acid WPC80 liquid) in the ratio 80:20. After the addition of lactose in the ratio 1:1.5 (protein/lactose), the solutions were freeze dried.

Heat treatment was applied at two different temperatures and durations as described elsewhere [29]. Briefly, for LT heated MP (LT-MP), the powder was heated for three weeks at 60 °C (a_w_ 0.23) and for HT heated MP (HT-MP) the powder was heated for 10 min at 130 °C (a_w_ 0.23). An unheated part of the milk powder was used as heating control (NT-MP).

### 2.3. Infant In Vitro Digestion

Simulated infant in vitro digestion was conducted in duplicate and was based on the protocol by Ménard et al. [30] with adaptations specific for the type of product described elsewhere. Compared to the adult digestion model, pH in the gastric phase (GP) was higher, while pH in the intestinal phase (IP) was lower. At the same time, enzyme concentrations were lower compared to the adult model [29]. Briefly, protein concentration of the meal was set to 1.2%. Digestion in the GP was conducted for 60 min with a pepsin activity set to 268 U/mL and at pH 5.3, but without the use of gastric lipase, as the milk powder contained <1% fat. Digestion in the IP was conducted for 60 min using pancreatin adjusted for its trypsin activity set to 16 U/mL digest and at pH 6.6. Samples were taken after 10 min and 60 min in the IP and stopped by the addition of 0.5 mM Pefabloc in the ratio Pefabloc/digest of 1/20 (*v*/*v*).

### 2.4. Caco-2 Cell Culture

The Caco-2 cell line was purchased from the American Type Culture Collection (Manassas, VA, USA), cultured in Dulbecco’s Modified Eagles Medium (DMEM) supplemented with 10% heat inactivated fetal bovine serum (FBS Hyclone) at 37 °C and in a humidified atmosphere containing 5% CO_2_. Cells were sub-cultured weekly upon confluence 85–95% using trypsination. Caco-2 cells were used from passage 30–40 and seeded into 24-well trans-wells (Greiner Bio-One, Kremsmünster, Austria) at a concentration of 0.225 × 10^6^ cells/mL in DMEM with 10% heat inactivated FBS. The medium was changed (apical (150 µL) and basolateral (700 µL)) every two–three days and cells were used for the transport experiment after 21 days of incubation. Before transport experiments, the transepithelial electrical resistance (TEER) value was measured and only wells with a TEER value higher than 750 Ω·cm^2^ were used.

### 2.5. Transport across the Caco-2 Cell Monolayer

Digest of one of the in duplicate in vitro digestions were diluted 1:1 with DMEM without phenol red, supplemented with 0.1% penicillin-streptomycin (10,000 U/mL) and applied to the apical side of the Caco-2 cell monolayer. TEER was measured at 37 °C using a Millicell-ERS Ώ Meter (Millipore, Molshein, France) and samples were incubated for 2 h with Caco-2 cells at 37 °C and 5% CO_2_ saturation. TEER was then measured and samples were taken from the basolateral side. Each sample was applied in duplicate. Samples were kept at −20 °C until further analysis.

### 2.6. Peptide Analysis

Digests after 10 min in the IP contained 3.6 mg/mL, 3.7 mg/mL, 3.6 mg/mL protein in NT-MP, LT-MP, and HT-MP, respectively. Digest after 60 min in the IP contained 4.2 mg/mL, 3.5 mg/mL, 3.8 mg/mL protein in NT-MP, LT-MP, and HT-MP, respectively. Samples were mixed 1:1 with trichloroacetic acid (20%) and centrifuged (10 min, 3500× *g*, 4 °C). The supernatants were cleaned using an in-house stage tip following a protocol described by Dingess et al. [31]. All samples were concentrated to compensate for the dilution during trichloroacetic acid precipitation.

Peptides were analysed on a Thermo nLC 1000 system (Thermo, Waltham, MA, USA) coupled to a LTQ orbitrap XL (Thermo Fisher Scientific, Breda, The Netherlands) for peptides in the in vitro digest, or Q Exactive HF-X X (Thermo Fisher Scientific, Breda, The Netherlands) for peptides on the basolateral side, as well as glycated peptides. Each sample was measured once. Chromatographic separation was conducted over a 0.10 × 250 mm ReproSil-Pur 120 C18-AQ 1.9 µm beads analytical column. A gradient consisting of acetonitrile in water spiked with 0.1% formic acid was used. Acetonitrile increased from 9% to 34% within 50 min using a flow rate of 0.5 µL/min. Full scan positive mode spectra (FTMS) were measured in the Orbitrap between m/z 380 and 1400 using high resolution (60,000). Collision-induced dissociation (LTQ) or Higher-energy collisional dissociation (Q Exactive HF-X) fragmentation was applied using an isolation width of 2 m/z and 1.2 m/z, respectively and 30 % and 24% normalized collision energy, respectively. MSMS scans were recorded in the data dependent mode for 2–3 2–5+charged peaks in the MS scan. For glycated peptides measured by the Q Exactive HF-X, a stepped collision energy (sCE 20-30-40) was used based on the method published by Liu et al. [32]. LC-MS/MS runs were processed using the MaxQuant version 1.6.3.4 with the Andromeda search engine [33]. Digestion mode was set to “unspecific”. A fixed modification was set for the formation of propionamide on cysteines, while variable modifications were set for acetylation of the peptide N-terminus, deamidation of asparagine and glutamine, and oxidation of methionine.

Peptides were identified using a bovine database from Uniprot (https://www.uniprot.org) that includes all the bovine milk proteins observed by Boggs et al. [34] (PRIDE PXD003011) in combination with a database for common contaminants. For peptide identification in MaxQuant with unspecific enzyme cleavage, peptides with a minimum length of 8 amino acids and maximum peptide length of 25 amino acids were identified to limit false identifications. Both peptide and protein false discovery rates were set to 1%. Post translational modifications were included for lactosylation (+324 Da), hexose modification (+162 Da), N^ε^-carboxymethyllysine modification (+58 Da), and pyrroline modification (+108 Da). For simplicity glucosyl lysine was used to refer to the hexose modification, although other hexoses could also result in this mass shift. Phosphorylated and glycated peptides were not included in the quantitation during the MaxQuant search. Due to the limited number of measurements, as well as the limitations in obtaining quantitative data from glycated peptides, data were reported as peptide count.

### 2.7. Data Analysis

Data were filtered for peptides derived from the six major milk proteins: α_s1_-casein, α_s2_-casein, β-casein, κ-casein, β-lactoglobulin, and α-lactalbumin. All peptides with score >80 were used for the overall peptide profiles, while for the sIgE binding epitopes and T-cell epitopes, only peptides with a score >100 were used. For total peptide count per sample in the digest, each duplicate digestion was filtered separately for non-modified peptides (intensity >0) and for phosphorylated and glycated peptides (identification by matching and/or by MS/MS). For all further analysis, only peptides identified in both duplicate digestions of the same heat treatment were reported.

### 2.8. sIgE Binding Epitope Identification

sIgE binding epitopes were identified by comparison of digestion-derived peptide sequences with sIgE binding epitopes as reviewed previously [35]. Peptides were reported as potential sIgE binding epitopes if their sequence matched ≥80% of the sequence of a known sIgE binding epitope.

### 2.9. T-Cell Epitope Prediction

T-cell epitopes were predicted using IEDB MHC Class II Binding Prediction software (http://tools.iedb.org/mhcii/, 02.06.2020) where an MHC class II allele reference set was obtained from (https://help.iedb.org/hc/en-us/articles/114094151851, 02.06.2020). The default method “IEDB recommended 2.2” was used for T-cell epitope predictions. All peptides within the size range 15–24 amino acids, which was previously reported as the size range for T-cell epitopes, were applied to the prediction software [36]. Peptides were reported as potential T-cell epitopes following the recommendations of the prediction tool, where each peptide reaching a percentile rank <10.0% can be considered as potential T-cell epitope.

## 3. Results

### 3.1. Identification of Peptides in In Vitro Digests

**Figure 1:**
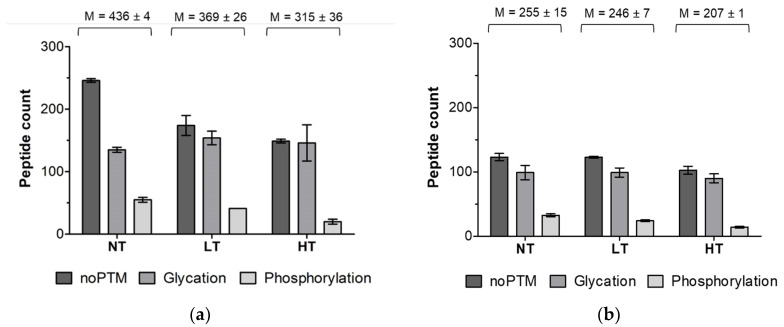
Total count of non-modified, glycated and phosphorylated digestion-derived peptides derived from cow’s milk protein. Samples were non-treated (NT), heated at low temperatures (LT), and heated at high temperature (HT) (**a**) after 10 min in the intestinal phase and (**b**) after 60 min in the intestinal phase. Number of peptides without post translational modification (noPTM), glycated, and phosphorylated peptides were compared. The minimum length for identification was eight amino acids. Error bars represent the standard deviation of duplicate digestions. The mean (M) of the total count of peptides per treatment and digestion time point ± standard deviation for duplicate digestions is shown above the bars.

Peptides released upon infant in vitro digestion after 10 and 60 min in the IP were analysed using LC-MS/MS. Only peptides derived from the six major MPs, α_s1_-casein, α_s2_-casein, β-casein, κ-casein, β-lactoglobulin, and α-lactalbumin were considered in the data analysis. Dry heating of MP decreased the number of peptides released upon infant in vitro digestion (Figure 1), where HT heating resulted in even less peptides than LT heating after 10 min (315 ± 36 vs. 369 ± 26 peptides) and 60 min (207 ± 1 vs. 246 ± 7 peptides) in the IP.

Differences in the modification state of the peptides (non-modified vs. glycated vs. phosphorylated peptides) were higher after 10 min than after 60 min in the IP. Heated samples showed comparable levels of glycated and non-modified peptides, while the NT-MP sample had two-fold more non-modified peptides than glycated peptides, after 10 min in the IP. At the same time, the number of phosphorylated peptides was 4.6-fold lower in HT-MP compared to NT-MP after 10 min in the IP, while LT-MP only showed a 1.6-fold decrease. This trend continued until 60 min in the IP, however to a lesser extent. Most peptides after 10 min in the IP were derived from β-casein, followed by β-lactoglobulin and α_s1_-casein, while a smaller number of peptides originated from α_s2_-casein, followed by κ-casein, and α-lactalbumin (Supplementary Materials: Figure S1a). This trend did not change after 60 min in the IP (Supplementary Materials: Figure S1b).

**Figure 2:**
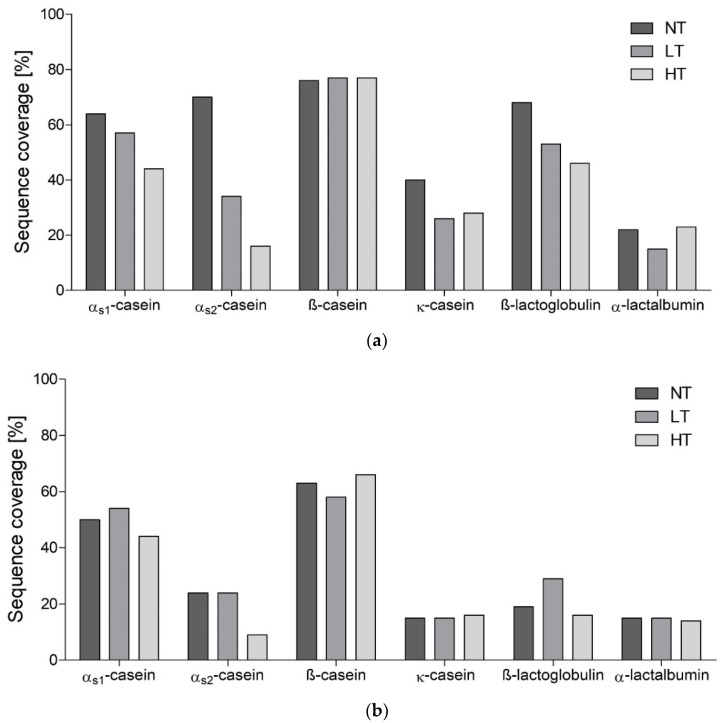
Sequence coverage of the six main milk proteins by digestion-derived peptides with and without posttranslational modification derived from in vitro digests of cow’s milk protein. Samples were non-treated (NT), heated at low temperature (LT), and heated at high temperature (HT) (**a**) after 10 min in the intestinal phase and (**b**) after 60 min in the intestinal phase.

In line with the number of peptides per protein, peptides generated after 10 min in the IP covered large parts of the protein sequences of β-casein, β-lactoglobulin, and α-caseins (Figure 2a).

Sequence coverage was higher for NT-MP compared to LT-MP and HT-MP. This difference was highest for the α-caseins and β-lactoglobulin, while β-casein showed no changes. After 60 min in the IP, only α_s2_-casein showed remarkably lower sequence coverage in HT-MP compared to NT-MP and LT-MP (Figure 2b). Due to the larger differences observed after 10 min in the IP, and the possibility of an immune response already at this stage of digestion, we focused mainly on the samples from 10 min in the IP.

**Figure 3:**
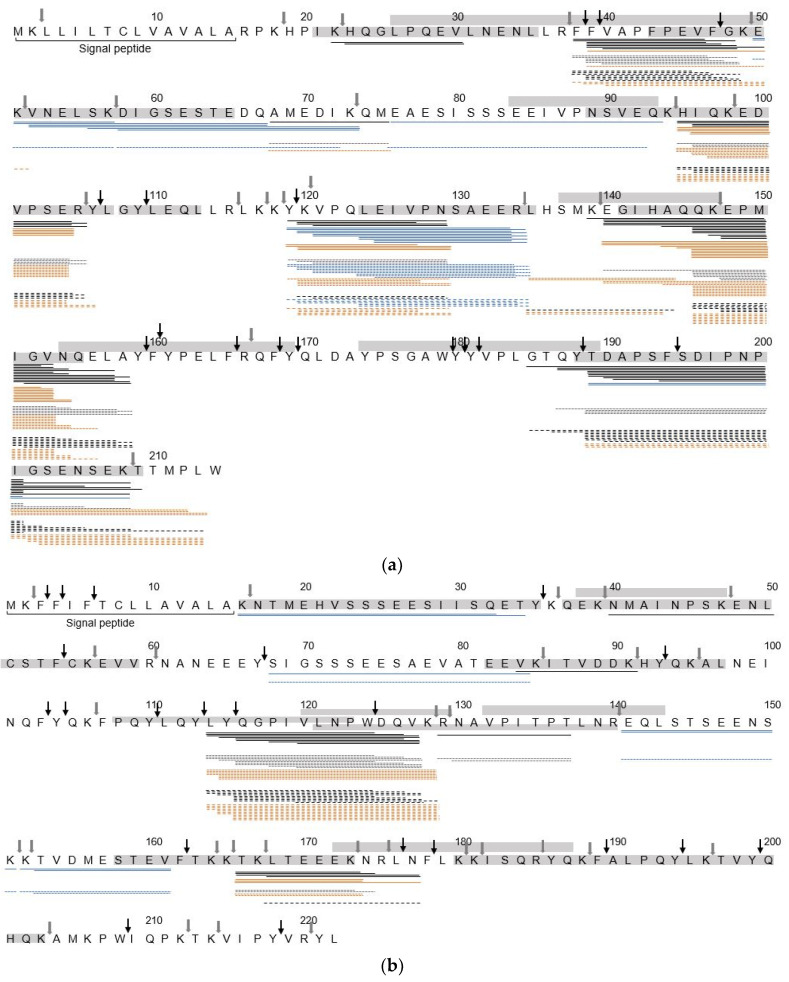

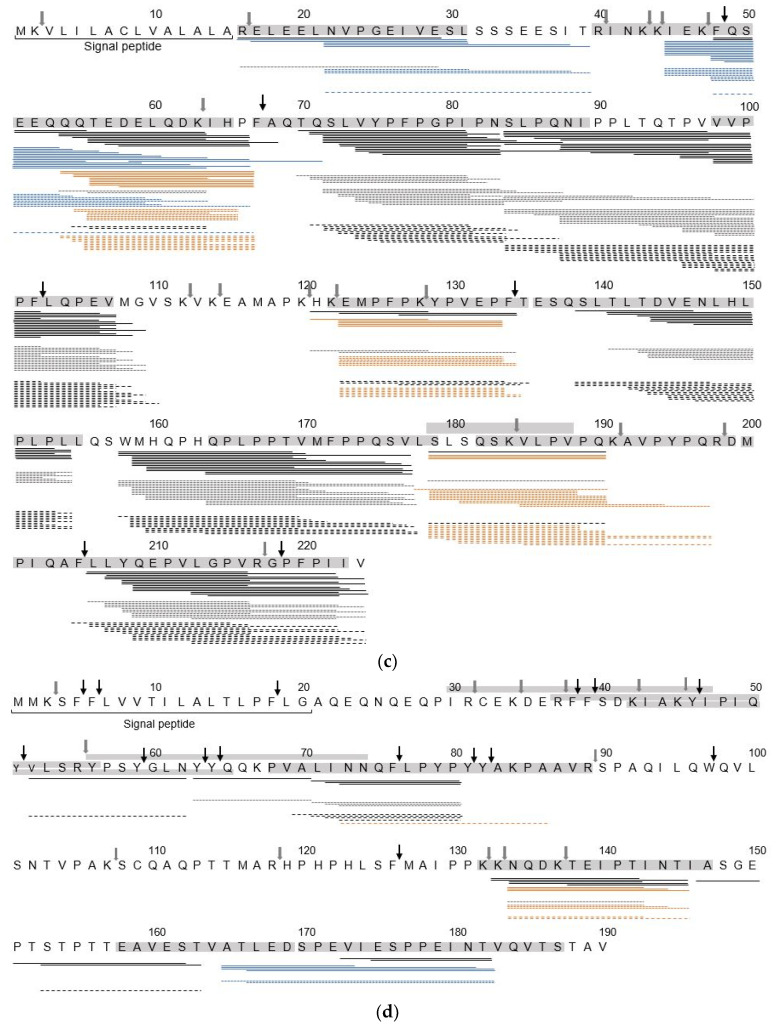

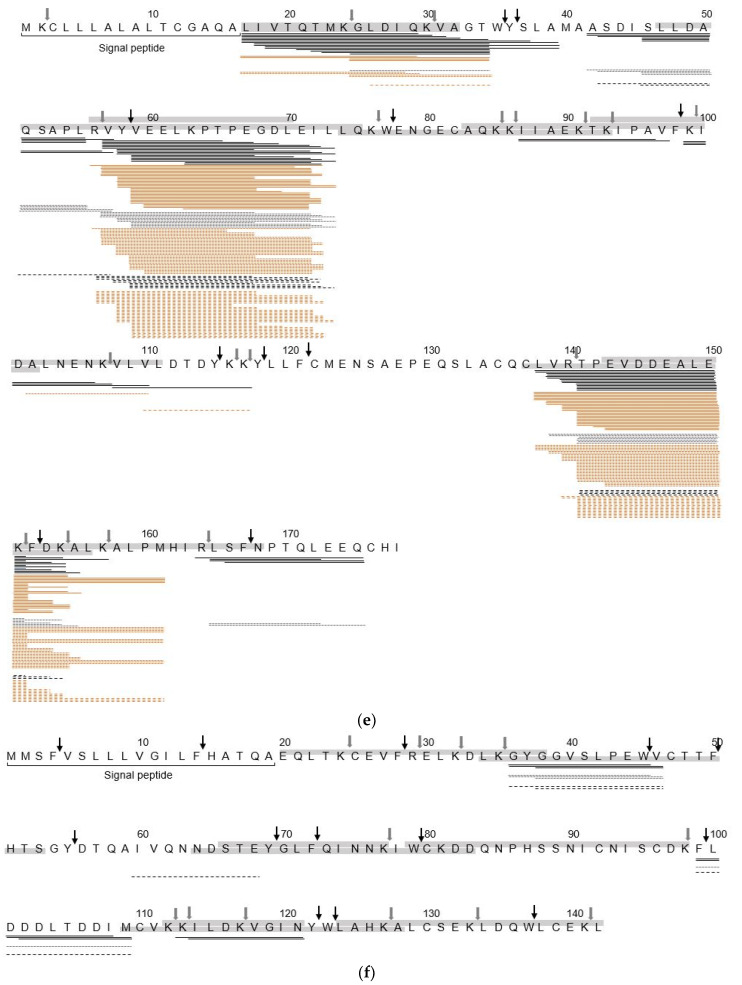
Sequence alignment of digestion-derived peptides identified after 10 min in the intestinal phase. Peptides derived from (**a**) α_s1_-casein, (**b**) α_s2_-casein, (**c**) β-casein, (**d**) κ-casein, (**e**) β-lactoglobulin, (**f**) α-lactalbumin identified after simulated infant in vitro digestion of non-treated cow’s milk protein (full line), heated at low temperature (dotted line), and heated at high temperature (dashed line). Glycated peptides (orange), phosphorylated peptides (blue), trypsin cleavage sites (thick grey down arrow), chymotrypsin cleavage sites (thin black down arrow). Trypsin and chymotrypsin cleavage sites were determined using Expasy Bioinformatics Resource Portal (https://web.expasy.org/peptide_cutter/last visited 08.06.2020).

In Figure 3, peptide sequence alignment is shown for peptides generated after 10 min in the IP. Independent from the heat treatment, all proteins showed specific regions that were similarly covered in all samples, but with different numbers of peptides generated in the same region.

In all proteins, the most differences in peptide distribution along the protein sequence were observed between HT-MP and the other samples. The peptides derived from β-lactoglobulin (Figure 3e) came from three main regions (f17–39, f56–73, and f138–161) and only minor differences were observed between heat treatments in f56–73, while in f17–39 and f138–161 much fewer peptides were found in HT-MP compared to NT-MP and LT-MP. In contrast, peptides derived from α_s2_-casein and β-casein where distributed all over the amino acid chain. Interestingly, most differences in caseins were observed as a result of the absence of phosphorylated peptides in HT-MP. In α_s1_-casein (Figure 3a), the region between f52–92 was mostly covered by phosphorylated peptides in NT-MP and LT-MP, while this region did not lead to the formation of peptides in HT-MP. Moreover, the number of peptides covering the same sequence part f52–92 was much lower in LT-MP than NT-MP. Similar observations, where the number of phosphorylated peptides was lower in at least one of the heated samples compared to NT-MP, were also made for α_s2_-casein f16–32, f68–84, f141–161(Figure 3b), β-casein f22–39 and f48–66 (Figure 3c), and κ-casein f165–182 (Figure 3d). In contrast, glycated peptides only had a minimal effect on differences in sequence coverage when comparing samples. In α_s1_-casein (Figure 3a), the regions f135–138 and f210–213 and in β-casein (Figure 3c) the region 191–197 were covered in LT-MP and HT-MP as a result of the presence of glycated peptides. The presence of glycated peptides, however, affected the number of peptides which arise from specific areas of the proteins. This was especially seen for the region f140–155 of α_s1_-casein in NT-MP, f113–128 of α_s2_-casein, and f179–197 of β-casein in both LT-MP and HT-MP.

While progressing intestinal digestion, only small changes were observed in the peptide alignment along the protein sequence of α_s1_-casein, β-casein, κ-casein, and α-lactalbumin (Supplementary Materials: Figure S2). In contrast, β-lactoglobulin showed two resistant areas f57–73 and f139–154 as well as α_s1_-casein at f119–134.

### 3.2. Identification of sIgE Epitopes and T-Cell Epitopes in the In Vitro Digest

sIgE binding epitopes were identified by comparison with known epitopes from the literature (Table 1) [35]. Peptides were reported as potential sIgE binding epitopes when at least 80% of the peptide sequence matched a known sIgE epitope sequence. Peptides derived from β-lactoglobulin contained 18 sIgE epitopes, followed by 16 derived from α_s1_-casein, 14 from β-casein, 3 from α_s2_-casein, and 1 from α-lactalbumin and κ-casein, respectively. The majority of sIgE epitopes were found in peptides derived from NT-MP; however, up to 69% of α_s1_-casein derived sIgE epitopes and 77% of β-casein derived sIgE epitopes were also found in either one or both heated samples. The peptides α_s1_-casein f189–213, α_s2_-casein f116–128, and β-casein f96–110 were only found in heated samples; however, their length only differed by a maximum of four amino acids from a similar peptide found in NT-MP and those four amino acids were not covering an additional sequence part of the sIgE binding epitope.

Additionally, glycated peptides that matched sequence parts of sIgE binding epitopes were identified after 10 min in the IP (Table 2). Most of such peptides were found in β-lactoglobulin; however, only five of them were exclusively found in heated samples and covered similar sequence parts as peptides that were also found in NT-MP. Glycated peptides matching the sequence of an sIgE binding epitope from α_s2_-casein and β-casein were exclusively found in heated samples. For α_s1_-casein, three out of four glycated peptides with sequence homology to an sIgE binding epitope were only found in HT-MP.

T-cell epitopes were predicted using IEDB MHC Class II Binding Prediction software. All modified and non-modified peptides that were predicted as potential T-cell binding epitopes are shown in Table 3.

Overall, 13 potential T-cell epitopes were found in the digest, with most epitopes deriving from α_s1_-casein and α_s2_-casein, followed by β-lactoglobulin, and β-casein. In the digest of NT-MP, 7 T-cell epitopes were found, of which five were also found in at least one of the heated samples. LT and HT heating resulted in the release of nine T-cell epitopes, respectively, with six solely found in heated samples. Of these, 40% were found in the digest of LT-MP and HT-MP were also glycated.

### 3.3. Peptides Identified at the Basolateral Compartment of the Caco-2 Cell Monolayer

**Figure 4:**
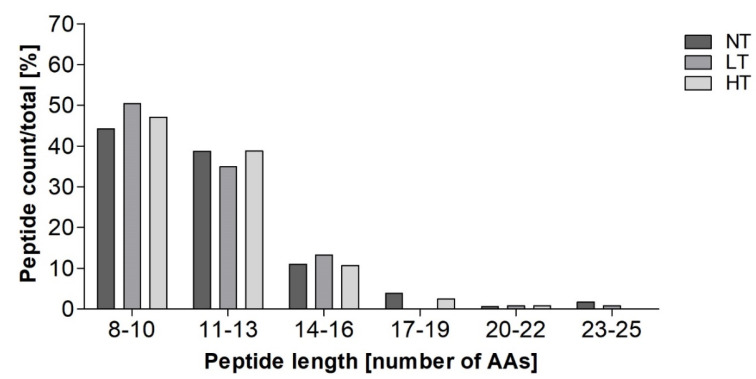
Peptide length distribution on the basolateral side of digestion-derived peptides, sampled after 10 min in the intestinal phase from simulated infant in vitro digests of cow’s milk protein, non-treated (NT), heated in the presence of lactose at low temperature (LT), and high temperature (HT), expressed as peptide count relative to the total number (NT: 181, LT: 129, HT: 121) of peptides in one sample.

To study the epithelial transport, in vitro digests sampled after 10 min in the IP were applied to a Caco-2 cell monolayer. The number of peptides found in the basolateral compartment for each sample decreased with heating intensity. Observed were 181, 129, and 121 peptides in NT-MP, LT-MP, and HT-MP, respectively. Moreover, most peptides were derived from α_s1_-casein, β-casein, and β-lactoglobulin (data not shown). Independent from the heat treatment, the majority of peptides were found in the size range between 8–10 and 11–13 amino acids (Figure 4). Compared to the composition in the digest before transport, relatively higher numbers of peptides in the size range between 8–10 and 11–13 were found (Figure 4 and Supplementary Materials: Figure S3). Interestingly, peptides up to 24 amino acids long were identified on the basolateral side of the Caco-2 cell monolayer, however at low numbers.

In NT-MP, less glycated peptides were found on the basolateral side (37%), compared to LT-MP (50%) and HT-MP (56%). This relative number of glycated peptides on the basolateral side increased in all samples compared to the digest before transport (35 ± 0%, 47 ± 1%, 49 ± 5% for NT-MP, LT-MP, and HT-MP, respectively). In all samples, the majority of those glycated peptides was modified to lactosyl lysine, followed by modification to glucosyl lysine, pyrroline and CML. Interestingly, the largest increase on the basolateral side was observed for the relative number of lactosyl lysine with 3%, 5%, and 5% increase and glucosyl lysine modified peptides with 5%, 7%, and 8% increase in NT-MP, LT-MP, and HT-MP, respectively (Supplementary Materials: Figure S3). This effect was larger in heated samples than in NT. HT-MP also showed 5% higher relative numbers of CML-modified peptides on the basolateral side, while the relative numbers of pyrroline-modified peptides shown were comparable to the digest.

### 3.4. sIgE Binding Epitopes on the Basolateral Side of the Caco-2 Cell Monolayer

Peptides identified at the basolateral side that carried at least 80% of the sequence of a known sIgE epitope are shown in Table 4. Similar to the observations in the digest (Table 2), most epitopes were found in peptides derived from β-lactoglobulin; however, only two of these peptides were unmodified. Moreover, only 19% of the glycated and non-glycated sIgE binding epitopes found in the digest (Table 1 and Table 2) were also found on the basolateral side (Table 4). Contrastingly, on average the total number of glycated and non-modified peptides found on the basolateral side corresponded to 49% of the number of glycated and non-modified peptides in the digest. Two of the peptides containing a sIgE epitope derived from α_s1_-casein (f52–67 and f123–133) were not identified in the digests before the Caco-2 cell experiment (Table 1). However, these peptides could derive from other precursor peptides (e.g., phosphorylated f56–67 and f124–133). Additionally, a peptide derived from β-lactoglobulin (f57–71) in non-modified and glycated form was previously found in all samples, while identification on the basolateral side was only possible in NT-MP.

From the T-cell epitopes identified in the digest, only one T-cell epitope derived from α_s1_-casein was found in HT-MP (f68–84). This data suggested an overall low passage of T-cell epitopes and sIgE binding epitope.

## 4. Discussion

### 4.1. Heat Treatment Dependent Differences in Peptide Profiles

Dry heated MP at LT and HT was subjected to simulated infant in vitro digestion and peptides were identified after 10 min and 60 min in the IP. As most differences between heat treatments in the digests were observed after 10 min in the IP, at which time the mucosal immune system in the gastrointestinal tract may already encounter antigens, the focus was on this digestion time point. Heat treatment of MP resulted in 15% and 28% less peptides released upon digestion after 10 min in the IP in LT-MP and HT-MP compared to NT-MP, while after 60 min in the IP only 3% and 19% less peptides were observed in LT-MP and HT-MP, respectively (Figure 1). The absence of peptides can be a result of both increased and impaired hydrolysis [7]. However, in our previous study we showed that HT dry heating impairs hydrolysis after 10 and 60 min in the IP suggesting that the absence of peptides results from decreased hydrolysis [29]. It is also possible that a larger pool of different linear and crosslinking MRPs can result in lower number of peptides as only the most abundant modifications were monitored. At the same time, the relative number of glycated peptides identified in the digest of heated samples was higher compared to NT-MP (Figure 1a). This is in line with the levels of CML and pentosidine that were reported previously for the samples used in this study which increased with increases in heating temperature [29]. Most of the peptides were modified to glucosyl lysine and lactosyl lysine (Supplementary Materials: Figure S4) and already a large proportion of lactoyslated peptides was observed in NT-MP. The comparison of peptide intensities, however, indicated that the quantities of glycated peptides in the heated samples were higher than in NT-MP (Supplementary Materials: Figure S5). This was in agreement with the findings of Milkovska-Stamenova et al. [37], who found 50 lactosylation sites in raw milk which increased to only 70–80 in ultra-high temperature treated milk. At the same time, quantification of the glycated peptides in their study showed much lower levels in raw milk compared to processed dairy products.

Most peptides were derived from α_s1_-casein, β-casein, and β-lactoglobulin (Supplementary Materials: Figure S1). In the two casein examples, this is probably related to the relatively higher concentration compared to the other proteins. For β-lactoglobulin, this is also related to the larger number of glycated peptides, of which each was counted as a separate peptide (Figure 3e). A heat treatment dependent decrease of sequence coverage after 10 min in the IP was especially observed for the two α-caseins and β-lactoglobulin (Figure 2a). For β-lactoglobulin, this originated from the absence or low number of peptides in the regions f17–39, f88–116, and f164–174 (Figure 3e). β-lactoglobulin, as a globular protein, is more sensitive to heating induced structural modifications compared to casein [38,39]. The regions f17–39 and f88–116 are rich in lysine residues, explaining the impairment of peptide generation especially in heated samples from this area via tryptic hydrolysis. The region f164–175 is located on the outside of the globular protein, partly incorporated in a β-strand and α-helix structure, which makes it rather easily accessible for digestive enzymes. However, it has been shown that upon heating in solution a α-β transition occurs, contributing to the aggregation of β-lactoglobulin via hydrophobic interactions [40]. This could explain the absence of peptides in the f164–175 region, as HT heating promotes the aggregation of β-lactoglobulin, but not in LT-MP and NT-MP. For α-caseins, a heating dependent decrease of sequence coverage was mainly reflected by the absence of phosphorylated peptides in HT-MP (Figure 3a,b) and is in line with the lower number of phosphorylated peptides (Figure 1a). Dephosphorylation has been reported upon heating in solution of caseinate at HT (140 °C) [41]. Both, hydrolysis of phosphoserine as well as β-elimination may induce dephosphorylation of casein. Michael addition subsequently to β-elimination and subsequent Michael addition results in protein crosslinking [41], which may also explain the lower number of peptides in HT-MP from sequence parts that are more phosphorylated. A study from Wada et al. [42] showed that dephosphorylation could decrease digestibility of heated dairy products. This is in line with the low digestibility of HT-MP observed in our previous study [29]. Next to digestibility, dephosphorylation could also decrease IgE binding capacity, indicating that overall IgE binding capacity to linear IgE binding epitopes could be lower for HT-MP [43]. In contrast, the region f22–39 of β-casein showed increasing number of phosphorylated peptides in HT while progressing digestion (Supplementary Materials: Figure S2c), indicating that in some cases a slower release of peptides could also be a possible explanation for the absence of phosphorylated peptides after 10 min in the IP. Glycated peptides resulted in a higher number of peptides in some areas (Figure 3a,c). This could possibly affect immunoreactivity if present on an epitope or by binding of these peptides to AGE receptors; however, it should be noted that quantities of glycated peptides were not measured and that it is not clear which effect the glycation of peptides has for epitope recognition. In summary, dry heating of MP decreases the number of peptides released upon simulated infant in vitro digestion and results in lower sequence coverage after 10 min in the IP. The discrepancies in sequence coverage of specific regions when comparing heat treatments can also be relevant for sIgE binding and T-cell epitope presentation. At the same time, the process of digestion results in fewer differences, suggesting that digestion kinetics are important determinants for differential release of immunoreactive digestion-derived peptides when comparing heat treatments.

### 4.2. Hydrolysis Resistant Areas

Most regions of κ-casein and α-lactalbumin from which peptides were released after 10 min in the IP (Figure 3d,f) were also detected after 60 min in the IP (Supplementary Materials: Figure S2). However, none of them were identified as areas of interest for possible immunological consequences. For α_s2_-casein, decreasing sequence coverage was only observed for NT-MP. This was related to the disappearance of the phosphorylated peptides (f16–34) and the peptide at f40–50, of which only low numbers were detected after 10 min in the IP. However, no potential epitopes were identified after 60 min in the IP. For α_s1_-casein and β-casein, sequence coverage and number of peptides showed only minor decrease with prolonged digestion (Figure 2 and Supplementary Materials: Figure S1). For β-casein, the peptide pattern between samples showed only minor differences and therefore also a comparable persistence of peptides carrying an sIgE binding epitope (f96–110, f123–134, and f164–177) (Supplementary Materials: Table S1). For α_s1_-casein, the region f202–213 was solely covered in heated samples by glycated peptides after 60 min in the IP, suggesting a higher digestion resistance of this area due to glycation. At the same time, f140–155 of α_s1_-casein, which has previously been reported to maintain high residual immunoreactivity after simulated in vitro digestion of spray dried milk powder [7], was only partly preserved until the end of the IP (Supplementary Materials: Figure S2a). A higher number of peptides in NT-MP-digests originating from this region could possibly result in a higher immunoreactivity of this sample. Next to this, a larger number of peptides in f119–134, which contained a potential T-cell epitope (Table 3) was still found at the end of intestinal digestion in all samples (Supplementary Materials: Figure S4a). In contrast to the two caseins, β-lactoglobulin showed large decreases of sequence coverage in all samples, related to the disappearance of f17–39 and f42–55. At the same time, the regions f57–73 and f139–154 of β-lactoglobulin were highly resistant to digestion until the end of intestinal digestion (Supplementary Materials: Figure S2e), independent from the heat treatment. This was in line with the findings by Egger at al. [44] who observed a high frequency of peptides within particularly these two areas of β-lactoglobulin until 120 min in the IP of a static in vitro model. Moreover, the findings for both β-lactoglobulin and α_s1_-casein were similar to previous findings by Picariello et al. [45], who described the sequence part f141–151 of β-lactoglobulin and f119–134 of α_s1_-casein as highly resistant to gastrointestinal digestion after simulated adult in vitro digestion. While there is no direct evidence for the presence of an immunoreactive structure within this region of β-lactoglobulin, f119–134 of α_s1_-casein partly covers the sequence of an sIgE binding epitope (Figure 3a) and was also identified as potential T-cell epitope (Table 3). Together with its high resistance until the end of intestinal digestion, this suggest a potential role of f119–134 in sIgE binding to the digest of MP, but independently from the heat treatment. To summarize these findings, caseins generally showed a higher resistance over large parts of their protein sequence until the end of gastrointestinal digestion, which was unaffected by the applied heat treatment. Therefore, no conclusions can be drawn from the resistance of specific areas within the protein sequence regarding differential immunoreactivity of dry heated MP compared to NT-MP.

### 4.3. Effect of Heat Treatment on Identification of IgE Binding Epitopes

Digestion-derived peptides were reported as potential sIgE binding epitopes if they covered at least 80% of the sequence of a linear sIgE binding epitope known from the literature [35]. The three proteins showing the highest numbers of digestion-derived peptides, α_s1_-casein, β-casein, and β-lactoglobulin (Supplementary Materials: Figure S1a) also led to the highest number of sIgE binding epitopes (Table 1) after 10 min in the IP. Most sIgE binding epitopes were found in the NT-MP digest, when compared to the heated samples (Table 1), which was in line with the higher number of peptides (Figure 1a) and the higher sequence coverage of NT-MP (Figure 2a). This suggests a higher availability of linear sIgE binding epitopes in NT-MP compared to dry heated MP. The opposite trend was observed for sIgE binding epitopes carrying a glycation side (Table 2). However, this trend did not continue until 60 min in the IP (Supplementary Materials: Table S1). After 60 min in the IP, most sIgE binding epitopes were found in the digest of NT-MP, of which the majority were, however, present in all samples. This can be explained by the overall smaller differences between samples with progressing digestion, which is possibly related to differences in digestion kinetics especially in the beginning of the IP. The effect of heating and glycation on sIgE binding has been subject to previous studies on isolated milk proteins or in mixture [15,16,17,19]. As reviewed by Nowak-Wegrzyn et al. [18], milk proteins show reduced sIgE binding upon extensive glycation via the MR. However, these observations are based on studies of undigested milk proteins and not of linear epitopes exclusively, and thus can probably not be extrapolated for all MRPs and peptides. A study by Gasparini et al. [46] reported an approach creating the basis for studying the effect of lactosylation on linear epitopes. However, data comparing sIgE binding of lactosylated vs. non-modified peptides is not available at this time. With respect to the predicted T-cell epitopes, ~50% were specifically found in the heated samples, but most of those peptides were glycated. To our knowledge only data on T-cell epitopes from α_s1_-casein, β-lactoglobulin, and α-lactalbumin are available in the literature. In a previous study it was shown that a peptide f118–135 of α_s1_-casein, which is similar to the T-cell epitope identified in our study (f119–135, Table 3) is recognized by 1 out of 10 cow’s milk allergic children. However, none of the major T-cell epitopes of α_s1_-casein identified in previous studies have been observed in the digests in our study [47,48,49]. The T-cell epitopes identified in β-lactoglobulin partly overlapped with the peptide sequences identified previously (f41–55) [50]. All T-cell epitopes from α_s1_-casein and β-lactoglobulin were predicted as ligands for multiple HLA alleles, indicating that their recognition could be less affected by individual differences in patients. While the majority of α_s1_-casein derived T-cell epitopes was phosphorylated, which in a previous study did not show consistent differences in epitope recognition [47], most of the α_s2_-casein derived T-cell epitopes were glycated (Table 3). To our knowledge there is no study directly showing the effect of glycation on T-cell epitope recognition. However, as glycation of food proteins has been shown to affect T-cell immunogenicity [21,51], it could be hypothesized that glycation of peptides matching a T-cell epitope could affect its immunogenicity. To summarize, dry heating of MP resulted in a lower number of peptides that match to known sIgE binding epitopes but a higher number of glycated sIgE binding epitopes. Moreover, T-cell epitopes were identified in the digest and equally distributed between samples, while glycated T-cell epitopes were solely found in heated samples. The consequences of glycation on sIgE epitope and T-cell epitope binding are, however, not clear.

### 4.4. Identification of Peptides on the Basolateral Side of the Caco-2 Cell Monolayer

Transport across the epithelial layer was assessed using a Caco-2 cell monolayer model. Peptide length distribution found on the basolateral side (Figure 4) indicated a favored transport of peptides up to 13 amino acids compared to the distribution in the digest (Supplementary Materials: Figure S3). Interestingly, peptides with a length up to 24 amino acids were also found on the basolateral side (Figure 4). The peptides in the larger size ranges (f17–19 and f20–22, and f23–25) were mainly non-glycated peptides derived from β-casein, which originated from hydrophobic patches within the sequence suggesting a passage via transcytosis [52]. Availability of larger peptides increases the possibility of recognition by the immune system. Moreover, transport of peptides carrying sIgE binding epitope sequences (e.g., f159–177) via transcytosis enables the peptide to reach the *lamina propria* intact, indicating the importance of also monitoring transport pathways when studying the availability of immunoreactive digestion-derived peptides. Most digestion-derived peptides on the basolateral side were derived from α_s1_-casein, β-casein, and β-lactoglobulin, which is probably related to the higher number of peptides in the digest (Supplementary Materials: Figure S2a). Consequently, sIgE binding epitopes found on the basolateral side were only identified for α_s1_-casein, β-casein, and β-lactoglobulin (Table 4). Moreover, sIgE binding epitopes were most abundant in NT-MP which was in line with the total number of digestion-derived peptides between samples (Table 1a) and presence amongst proteins (Supplementary Materials: Figure S1a). However, only 19% of the sIgE binding epitopes (non-modified and glycated) and one T-cell epitope identified in the digests were also found on the basolateral side (Table 1, Table 2 and Table 4), while on average 49% of the number of peptides in the digest were found on the basolateral side, suggesting some sort of epitope-excluding effect of the epithelial layer. For T-cell epitopes it could be hypothesized that this was related to size, as the size ranges 8–10 as well as 11–13 were preferably transported, while T-cell epitopes normally have a length between 15–24 amino acids [36]. In contrast, most sIgE binding epitopes were identified within the smaller size ranges. Next to peptide size, the transport across the Caco-2 cell monolayer can also be determined by charge and hydrophobicity [52]. However, further studies would be necessary to determine peptide properties to find the reasons for the observed restriction of epitope transport across the Caco-2 cell monolayer. Moreover, it should be noted that in vivo a larger number of M-cells as well as specialized dendritic cells are present in the small intestine, that are able to directly sample antigens from the intestinal lumen [53]. It is thus hypothesized that the translocation of IgE and T-cell epitopes in vivo could be directed towards specialized cells rather than transport via normal enterocytes.

**Figure 5:**
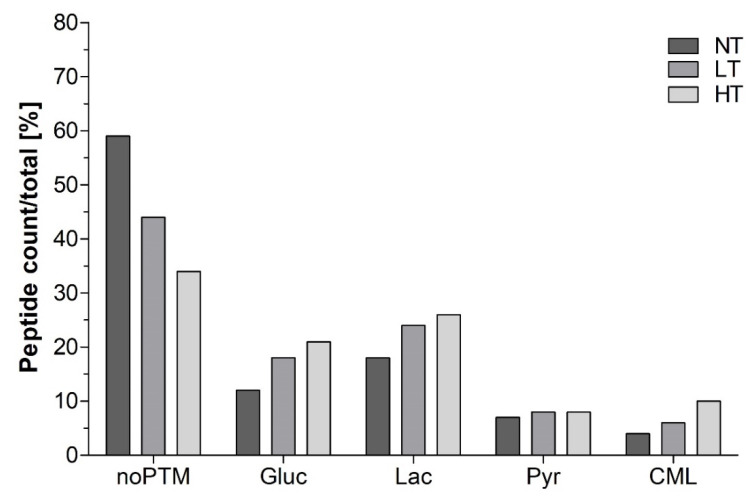
Digestion-derived peptides identified on the basolateral side of Caco-2 cells exposed to in vitro digests of cow’s milk protein, non-treated (NT), heated in the presence of lactose at low temperature (LT), and high temperature (HT). Peptides without posttranslational modification (noPTM), as well as modification to glucosyl lysine (Gluc), lactosyl lysine (Lac), pyrroline (Pyr), and carboxymethyl lysine (CML) are shown expressed as peptide count relative to the total number (NT: 181, LT: 129, HT: 121) of peptides in one sample.

In contrast to this, transport of a relatively higher number of glycated peptides was observed on the basolateral side, e.g., in dry heated samples, compared to the composition in the digest. Moreover, data suggested a possible preference for the transport of lactosyl lysine and glucosyl lysine-modified peptides amongst all samples, as the percentage of these peptides showed an increasing trend on the basolateral side compared to the digest (Supplementary Materials: Figures S4 and S5). As reviewed by Moradi et al. [54], N- and O-glycosylation with different mono- and polysaccharides of therapeutic peptides has been shown to increase their transport across various biological membranes including Caco-2 cells. For example, Varamini et al. [55] observed a 700-fold increased transport across the Caco-2 cell monolayer after modification of the N-terminal amino group from endomorphin-1 with lactose and suggested that this transport took place via a lactose-selective transporter. Such transporter-mediated translocation could be a possible explanation for the facilitated migration of glucosyl lysine and lactosyl lysine-modified digestion-derived peptides across the Caco-2 cell monolayer. However, it should be noted that the position and type of linkage (N- or O-linked) can strongly affect the structure, functionality and transporter mediated uptake of the peptides [54,56]. Therefore, an extrapolation of these findings to any peptide and any kind of modification is probably not possible. With respect to the potential immunological consequences, it is suggested that glycation if present on a linear sIgE binding epitope can affect the interaction between the peptide and the antibody [46]. Moreover, AGEs themselves have also been reported to modulate inflammatory pathways by binding to receptors for AGEs [14]. For the example of peptide-bound CML, it has been shown that it is a potent ligand for the receptor for AGEs and thus possibly affects inflammatory pathways [20]. This study showed that glycated peptides larger than 7 amino acids are transported independent of the type of modification (Figure 5). The findings of this study suggested that diets with high AGE content can also result in higher uptake of AGE-modified peptides. As recently shown, the binding of AGE receptors depends on the concentration of food protein bound AGEs [22]. Therefore, quantitative data would be necessary to better judge the impact of the transport of AGE-modified peptides on the gastrointestinal immune system as well as the involved transport pathways. To summarize, results indicated that several potentially immunoreactive peptides are transported across a model epithelial barrier. In general, the presence of peptides on the basolateral side is more affected by the overall composition of the digest rather than the selective transport of specific peptides. Nevertheless, transport seemed to be favored for smaller peptides (up to 13 amino acids) as well as peptides modified to lactosyl lysine and glucosyl lysine. This should, however, be further investigated using quantitative data on selected modified vs. non-modified peptides. At the same, time transport of sIgE binding epitopes and T-cell epitopes was limited, which is possibly related to some intrinsic properties of these peptides.

This study aimed to give an overview of the composition and transport of peptides derived after simulated infant in vitro digestion of differentially dry heated MP. However, this also resulted in some limitations, as only qualitative data was presented and allergenicity as well as immunogenicity was not measured directly. Moreover, other structures that could affect immunogenicity as well as allergenicity, such as aggregated protein that might also resist in vitro digestion, have not been considered [57,58].

## 5. Conclusions

This study showed that different peptide profiles are generated during simulated infant in vitro digestion of milk that was dry heated in the presence of lactose. HT dry heating had the largest effects on peptide generation, resulting in much lower numbers of peptides and a lower sequence coverage. Moreover, a much lower number of sIgE binding epitopes but a larger proportion of glycated sIgE binding epitopes and T-cell epitopes in heated samples indicated that immunogenicity and allergenicity of these samples could be affected. However, this needs to be further tested. Transport studies showed that the transport of sIgE epitopes and T-cell epitopes across the Caco-2 cell monolayer is limited, highlighting the importance of evaluating different transport pathways. It is hypothesized that transport of lactosyl lysine and glucosyl lysine-modified peptides was favored, while CML and pyrroline-modified peptides were transported depending on their presence in the digest. This resulted in relatively more glycated peptides on the basolateral side in heated samples, indicating that if the initial level of MR is high, this will also increase the transport of glycated peptides and can thereby possibly affect immunoreactivity via interaction with AGE receptors. This pointed out the importance of studying the effect of glycation on the peptide level on immunogenicity and allergenicity.

## Figures and Tables

**Table 1:** sIgE binding epitopes ^1^ identified in digestion-derived peptides after 10 min in the intestinal phase. Peptides were identified in cow’s milk protein, non-treated (NT), heated at low temperature (LT), and heated at high temperature (HT), after simulated infant in vitro digestion and derived from casein (cn), β-lactoglobulin (lg), and α-lactalbumin (lac). Peptides matching exactly the sIgE binding epitope sequence are indicated (*). Amino acids (AAs) position indicates the position within the proteins including the signal peptide. Peptides carrying a post translational modifications (PTM) are marked with phosphorylation (Phos), whereas phosphorylated serine (S) and threonine (T) residues are highlighted in bold and underline.

Protein	Sample	Peptide Sequence	AAs Position	sIgE Epitope AAs Position	PTM	
α_s1_-cn	NT, LT, HT	VNEL**S**KDIG**S**E**ST**EDQ	52–67	54–63	Phos	
	NT, LT, HT	VNEL**S**KDIG**S**E**ST**EDQAMEDIK	52–73	54–63	Phos	
	NT, LT, HT	KVPQLEIVPN**S**AEE	120–133	124–135	Phos	
	NT, LT, HT	KVPQLEIVPN**S**AEER	120–134	124–135	Phos	
	NT, LT	VPQLEIVPN**S**AEER	121–134	124–135	Phos	
	NT, LT, HT	LEIVPN**S**AEE	124–133	124–135	Phos	
	NT, LT, HT	LEIVPN**S**AEER	124–134	124–135	Phos	
	NT	EIVPN**S**AEER	125–134	124–135	Phos	
	NT	KEGIHAQQKEPMIGV	139–153	137–147	N/A	
	NT	EGIHAQQKEPMIGV	140–153	141–155	N/A	
	NT, HT	GTQYTDAPSFSDIPNPI	185–201	186–200	N/A	
	NT, LT, HT	QYTDAPSFSDIPNPI	187–201	186–200	N/A	
	NT	QYTDAPSFSDIPNPIGSENSEK	187–208	188–209	N/A	
	NT, LT, HT	TDAPSFSDIPNPIGSENSEK	189–208	188–209	N/A	
	NT,LT,HT	TDAP**S**F**S**DIPNPIG**S**EN**S**EK	189–208	188–209	Phos	
	NT	TDAPSFSDIPNPIGSENSEKT	189–209	188–209	N/A	
α_s2_-cn	NT	KNTMEHV**SSS**EE**S**IISQ	16–32	16–35	Phos	
	NT	KNTMEHV**SSS**EE**S**IISQET	16–34	16–35	Phos	
	HT	QGPIVLNPWDQVK	116–128	120–129	N/A	
β-cn	NT, LT	RELEELNVPGEIVE	16–29	16–31	N/A	
	NT	RELEELNVPGEIVE**S**L	16–31	16–31	Phos	*
	NT	ELEELNVPGEIVE**S**L	17–31	16–31	Phos	
	NT	TEDELQDKIHPFA	56–68	60–69	N/A	
	NT, LT, HT	SLVYPFPGPIPNS	72–84	70–85	N/A	
	NT, LT, HT	PVVVPPFLQPE	96–106	98–107	N/A	
	NT, LT, HT	PVVVPPFLQPE	96–107	98–107	N/A	
	NT, LT, HT	PVVVPPFLQPEVMG	96–109	98–107	N/A	
	LT, HT	PVVVPPFLQPEVMGV	96–110	98–107	N/A	
	NT, LT, HT	VVPPFLQPE	98–106	98–107	N/A	
	NT, LT, HT	VVPPFLQPEV	98–107	98–107	N/A	*
	NT, LT, HT	EMPFPKYPVEPF	123–134	122–135	N/A	
	NT, LT	QPLPPTVMFPPQS	164–176	164–179	N/A	
	NT, LT, HT	QPLPPTVMFPPQSV	164–177	164–179	N/A	
κ-cn	NT	KNQDKTEIPTINT	133–145	132–147	N/A	
β-lg	NT	LIVTQTMKGLDIQ	17–29	17–32	N/A	
	NT	LIVTQTMKGLDIQKV	17–31	17–32	N/A	
	NT	LIVTQTMKGLDIQKVA	17–32	17–32	N/A	
	NT	LIVTQTMKGLDIQKVAGT	17–34	17–32	N/A	
	NT	LIVTQTMKGLDIQKVAGTWYS	17–37	17–32	N/A	
	NT	LIVTQTMKGLDIQKVAGTWYSLA	17–39	17–32	N/A	
	NT	IVTQTMKGLDIQKVAGT	18–34	17–32	N/A	
	NT	IVTQTMKGLDIQKVAGTWYSLA	18–39	17–32	N/A	
	NT	VTQTMKGLDIQKVAGT	19–34	17–32	N/A	
	NT	VTQTMKGLDIQKVAGTWYSLA	19–39	17–32	N/A	
	NT, LT, HT	VYVEELKPTPEGDLE	57–71	56–70	N/A	
	NT, LT, HT	VYVEELKPTPEGDLEI	57–72	56–70	N/A	
	NT, LT	VYVEELKPTPEGDLEIL	57–73	56–70	N/A	
	NT, LT, HT	YVEELKPTPEGDLE	58–71	56–70	N/A	
	NT, LT, HT	YVEELKPTPEGDLEI	58–72	56–70	N/A	
	NT, LT	YVEELKPTPEGDLEIL	58–73	56–70	N/A	
	NT	LVRTPEVDDEALEK	138–151	136–150	N/A	
	NT	LVRTPEVDDEALEKFDK	138–154	137–156	N/A	
α-lac	NT	KILDKVGIN	113–121	112–121	N/A	

^1^ Peptides were reported as sIgE binding epitopes if their sequence contained at least 80% of the sequence of an sIgE binding epitope.

**Table 2:** sIgE binding epitopes ^1^ identified in glycated digestion-derived peptides after 10 min in the intestinal phase. Peptides were identified in cow’s milk protein, non-treated (NT), heated at low temperature (LT), and heated at high temperature (HT), after simulated infant in vitro digestion and derived from casein (cn) and β-lactoglobulin (lg). Amino acids (AAs) position indicates the position within the proteins including the signal peptide. Peptides containing post translational modification (PTM) to lactosyl lysine (Lac), glucosyl lysine (Gluc), N^ε^-carboxymethyllysine (CML), and pyrroline (Pyr) on lysine (K) are indicated, and modified K residues are highlighted in bold and underlined with modification site probability given in brackets if multiple options were identified.

Protein	Sample	Peptide Sequence	AAs Position	sIgE Epitope AAs Position	PTM
α_s1_-cn	NT, LT	EGIHAQQ**K**EPMIGV	140–153	141–155	Lac
	HT	TDAPSFSDIPNPIGSENSE**K**	189–208	188–209	Lac
	HT	TDAPSFSDIPNPIGSENSE**K**TTMPL	189–213	188–209	Gluc
	HT	TDAPSFSDIPNPIGSENSE**K**TTMPL	189–213	188–209	Lac
	NT, LT	EGIHAQQ**K**EPMIGV	140–153	141–155	Lac
α_s2_-cn	LT, HT	LYQGPIVLNPWDQV**K**	114–128	120–129	Lac
	LT, HT	LYQGPIVLNPWDQV**K**	114–128	120–129	Gluc
	LT, HT	LYQGPIVLNPWDQV**K**	114–128	120–129	CML
	LT, HT	LYQGPIVLNPWDQV**K**	114–128	120–129	Pyr
	LT, HT	YQGPIVLNPWDQV**K**	115–128	120–129	Lac
	LT, HT	YQGPIVLNPWDQV**K**	115–128	120–129	Gluc
	LT, HT	YQGPIVLNPWDQV**K**	115–128	120–129	CML
	HT	QGPIVLNPWDQV**K**	116–128	120–129	Lac
	HT	QGPIVLNPWDQV**K**	116–128	120–129	Gluc
	HT	QGPIVLNPWDQV**K**	116–128	120–129	CML
	HT	QGPIVLNPWDQV**K**	116–128	120–129	Pyr
β-cn	LT, HT	EMPFP**K**YPVEPF	123–134	122–135	Lac
	LT, HT	EMPFP**K**YPVEPF	123–134	122–135	Gluc
	HT	SLSQS**K(1)**VLPVPQ**K(1)**AVPYPQ	179–197	182–199	Lac
β-lg	LT, HT	LIVTQTM**K**GLDIQ	17–29	17–32	Lac
	NT	LIVTQTM**K(1)**GLDIQ**K(1)**VAGT	17–34	17–32	Lac
	LT	RVYVEEL**K**PTPEGDLE	56–71	56–70	Lac
	NT	RVYVEEL**K**PTPEGDLEI	56–72	56–70	Lac
	NT	VYVEEL**K**PTPEGDLE	57–70	56–70	Lac
	NT, LT, HT	VYVEEL**K**PTPEGDLE	57–71	56–70	Lac
	NT, LT, HT	VYVEEL**K**PTPEGDLE	57–71	56–70	Gluc
	NT, LT, HT	VYVEEL**K**PTPEGDLE	57–71	56–70	CML
	NT, LT, HT	VYVEEL**K**PTPEGDLE	57–71	56–70	Pyr
	NT, LT, HT	VYVEEL**K**PTPEGDLEI	57–72	56–70	Lac
	NT, LT, HT	VYVEEL**K**PTPEGDLEI	57–72	56–70	Gluc
	NT, LT, HT	YVEEL**K**PTPEGDLE	58–71	56–70	Lac
	NT, LT, HT	YVEEL**K**PTPEGDLE	58–71	56–70	Gluc
	NT, LT, HT	YVEEL**K**PTPEGDLE	58–71	56–70	CML
	NT, LT, HT	YVEEL**K**PTPEGDLE	58–71	56–70	Pyr
	LT, HT	YVEEL**K**PTPEGDLEI	58–72	56–70	Lac
	LT, HT	YVEEL**K**PTPEGDLEI	58–72	56–70	Gluc
	LT, HT	YVEEL**K**PTPEGDLEI	58–72	56–70	CML
	NT, HT	YVEEL**K**PTPEGDLEIL	58–73	56–70	Lac
	NT	LVRTPEVDDEALE**K(1)**FD**K(1)**	138–154	137–156	Lac
	NT	LVRTPEVDDEALE**K(1)**FD**K(1)**	138–154	137–156	Pyr
	NT, LT	LVRTPEVDDEALE**K(1)**FD**K(1)**AL**K(1)**ALPM	138–161	137–156	Lac
	NT, LT	LVRTPEVDDEALEKFD**K(0.8)**AL**K(0.2)**ALPM	138–161	137–156	Gluc
	NT, LT	LVRTPEVDDEALE**K(1)**FDKALKALPM	138–161	137–156	CML
	NT, LT	LVRTPEVDDEALE**K(1)**FD**K(1)**ALKALPM	138–161	137–156	Pyr

^1^ Peptides were reported as sIgE binding epitopes if their sequence contained at least 80% of the sequence of an sIgE binding epitope.

**Table 3:** Potential T-cell epitopes identified after 10 min in the intestinal phase. Peptides were identified as potential T-cell epitopes using IEDB MHC Class II Binding Prediction software (http://tools.iedb.org/mhcii/). Digestion-derived peptides identified from cow’s milk protein, non-treated (NT), dry heated at low temperature (LT), and dry heated at high temperature (HT) applied to simulated infant in vitro digestion, derived from casein (cn) and β-lactoglobulin (lg). Peptides matching exactly the sIgE binding epitope sequence are indicated (*). Amino acids (AAs) position indicates the position within the proteins including the signal peptide. Unmodified peptides and peptides with post translational modifications (PTM), via phosphorylation (Phos), as well as modification to glucosyl lysine (Gluc), lactosyl lysine (Lac), N^ε^-carboxymethyllysine (CML), and pyrroline (Pyr) were reported. Modified amino acids are highlighted in bold and underlined.

Protein	Sample	Sequence	HLA-Allele	AAs Position	PTM	Perc. Rank
α_s1_-cn	NT, LT	EAESI**SSS**EEIVPNSVEQ	HLA-DQA1*03:01/DQB1*03:02;	76–93	Phos	2.5
			HLA-DQA1*04:01/DQB1*04:02			5.3
	NT, LT	YKVPQLEIVPN**S**AEE	HLA-DRB1*04:05;	119–133	Phos	1.9
			HLA-DQA1*04:01/DQB1*04:02;			4.1
			HLA-DQA1*03:01/DQB1*03:02			5.8
	NT, LT, HT	YKVPQLEIVPN**S**AEER	HLA-DRB1*04:05;	119–134	Phos	2.2
			HLA-DQA1*04:01/DQB1*04:02;			5.9
			HLA-DQA1*03:01/DQB1*03:02			6.5
	NT, LT, HT	KVPQLEIVPN**S**AEER	HLA-DRB1*04:05;	120–134	Phos	1.9
			HLA-DQA1*04:01/DQB1*04:02;			5.6
			HLA-DQA1*03:01/DQB1*03:02			5.9
α_s2_-cn	NT, LT, HT	SIGSSSEESAEVATEEV	HLA-DQA1*04:01/DQB1*04:02;	68–84	n.a.	0.14
			HLA-DQA1*03:01/DQB1*03:02			0.18
	LT, HT	LYQGPIVLNPWDQV**K**	HLA-DRB1*13:02	114–128	Gluc	9.7
	LT, HT	LYQGPIVLNPWDQV**K**	HLA-DRB1*13:02	114–128	Lac	9.7
	LT, HT	LYQGPIVLNPWDQV**K**	HLA-DRB1*13:02	114–128	CML	9.7
	LT, HT	LYQGPIVLNPWDQV**K**	HLA-DRB1*13:02	114–128	Pyr	9.7
β-cn	HT	SLTLTDVENLHLPLP	HLA-DPA1*03:01/DPB1*04:02	139–153	N/A	6.3
β-lg	NT	VTQTMKGLDIQKVAGT	HLA-DRB4*01:01	19–34	N/A	7.9
	NT	ASDISLLDAQSAPLRV	HLA-DRB4*01:01;	42–57	N/A	4.0;
			HLA-DRB1*01:01;			6.6;
			HLA-DRB1*13:02;			7.9;
			HLA-DRB1*12:01			8.2;
			HLA-DQA1*03:01/DQB1*03:02			9
	HT	SDISLLDAQSAPLRV	HLA-DRB4*01:01;	43–57	N/A	3.3
			HLA-DRB1*01:01;			4.4
			HLA-DRB1*12:01;			6.2
			HLA-DRB1*13:02;			6.3
			HLA-DRB1*09:01			7.2

**Table 4:** Digestion-derived peptides covering sIgE binding epitope sequences ^1^, identified on the basolateral side of the Caco-2 cell monolayer. Peptides were generated after digestion of cow’s milk protein, non-treated (NT), heated at low temperature (LT), and heated at high temperature (HT), in an infant in vitro model. Amino acids (AAs) position indicates the position within the proteins including the signal peptide. Peptides derived from α_s1_-casein (α_s1_-cn), β -casein (β-cn), and β-lactoglobulin (β-lg). Peptides with and without post translational modification (PTM) to lactosyl lysine (Lac), glucosyl lysine (Gluc), N^ε^-carboxymethyllysine (CML), and pyrroline (Pyr) are shown. Digestion-derived peptides covering the exact sequence of a sIgE binding epitope are indicated with *.

Protein	Sample	Peptide Sequence	AAs Position	sIgE Epitope AAs Position	PTM	
α_s1_-cn	NT, LT	VNELSKDIGSESTEDQ	52–67	54–63	N/A	
	NT, LT	KVPQLEIVPNSAEE	120–133	124–135	N/A	
	NT, LT	QLEIVPNSAEE	123–133	124–135	N/A	
	NT, LT, HT	LEIVPNSAEER	124–134	124–135	N/A	
β-cn	NT, LT	PVVVPPFLQPEV	96–107	98–107	N/A	
	NT, LT, HT	VVPPFLQPE	98–106	98–107	N/A	
	NT, LT, HT	VVPPFLQPEV	98–107	98–107	N/A	*
β-lg	NT, LT, HT	VYVEELKPTPEGDLE	57–71	56–70	N/A	
	NT	VYVEEL**K**PTPEGDLE	57–71	56–70	CML	
	NT	VYVEEL**K**PTPEGDLE	57–71	56–70	Lac	
	NT	VYVEEL**K**PTPEGDLE	57–71	56–70	Pyr	
	NT, HT	YVEELKPTPEGDLE	58–71	56–70	N/A	
	NT, LT, HT	YVEEL**K**PTPEGDLE	58–71	56–70	CML	
	NT, LT, HT	YVEEL**K**PTPEGDLE	58–71	56–70	Lac	
	NT, LT, HT	YVEEL**K**PTPEGDLE	58–71	56–70	Pyr	

^1^ Peptides were reported as sIgE binding epitopes if their sequence contained at least 80% of the sequence of an sIgE binding epitope.

## Supplementary Materials

The following are available online at https://www.mdpi.com/2072-6643/12/8/2483/s1, Figure S1: Peptides derived from each of the five major milk proteins after infant in vitro digestion of cow’s milk protein, Figure S2: Sequence alignment of digestion-derived peptides after 60 min in the intestinal phase, Figure S3: Peptide length distribution identified in the in vitro digests of cow’s milk protein after 10 min in the intestinal phase, Figure S4: Non-modified vs. glycated digestion-derived peptides identified after 10 min in the intestinal phase, Figure S5: Summed intensities of peptides associated with a specific modification, within the same amino acid sequence. Table S1: sIgE binding epitopes ^1^ identified in digestion-derived peptides derived from cow’s milk protein after 60 min in the intestinal phase, Table S2: Potential T-cell epitopes identified after 60 min in the intestinal phase.

## References

[1] Kim J.S., Nowak-Wgrzyn A., Sicherer S.H., Noone S., Moshier E.L., Sampson H.A. (2011). Dietary baked milk accelerates the resolution of cow’s milk allergy in children. J. Allergy Clin. Immunol..

[2] Van Boekel M.A.J.S. (1998). Effect of heating on Maillard reactions in milk. Food Chem..

[3] Arena S., Renzone G., D’Ambrosio C., Salzano A.M., Scaloni A. (2017). Dairy products and the Maillard reaction: A promising future for extensive food characterization by integrated proteomics studies. Food Chem..

[4] Birlouez-Aragon I., Pischetsrieder M., Leclère J., Morales F.J., Hasenkopf K., Kientsch-Engel R., Ducauze C.J., Rutledge D. (2004). Assessment of protein glycation markers in infant formulas. Food Chem..

[5] Pischetsrieder M., Henle T. (2012). Glycation products in infant formulas: Chemical, analytical and physiological aspects. Amino Acids.

[6] Dupont D., Mandalari G., Mollé D., Jardin J., Rolet-Répécaud O., Duboz G., Léonil J., Mills C.E.N., Mackie A.R. (2010). Food processing increases casein resistance to simulated infant digestion. Mol. Nutr. Food Res..

[7] Dupont D., Boutrou R., Menard O., Jardin J., Tanguy G., Schuck P., Haab B.B., Leonil J. (2010). Heat treatment of milk during powder manufacture increases casein resistance to simulated infant digestion. Food Dig..

[8] Sánchez-Rivera L., Ménard O., Recio I., Dupont D. (2015). Peptide mapping during dynamic gastric digestion of heated and unheated skimmed milk powder. Food Res. Int..

[9] Kopf-Bolanz K.A., Schwander F., Gijs M., Vergères G., Portmann R., Egger L. (2014). Impact of milk processing on the generation of peptides during digestion. Int. Dairy J..

[10] Torcello-Gómez A., Dupont D., Jardin J., Briard-Bion V., Deglaire A., Risse K., Mechoulan E., Mackie A. (2020). The pattern of peptides released from dairy and egg proteins is highly dependent on the simulated digestion scenario. Food Funct..

[11] Wada Y., Phinney B.S., Weber D., Lönnerdal B. (2017). In vivo digestomics of milk proteins in human milk and infant formula using a suckling rat pup model. Peptides.

[12] Zenker H.E., Van Lieshout G.A.A., Van Gool M.P., Bragt M.C.E., Hettinga K.A. (2020). Lysine blockage of milk proteins in infant formula impairs overall protein digestibility and peptide release. Food Funct..

[13] Zhao D., Li L., Le T.T., Larsen L.B., Xu D., Jiao W., Sheng B., Li B., Zhang X. (2019). Digestibility of glycated milk proteins and the peptidomics of their in vitro digests. J. Sci. Food Agric..

[14] Teodorowicz M., Van Neerven J., Savelkoul H. (2017). Food processing: The influence of the maillard reaction on immunogenicity and allergenicity of food proteins. Nutrients.

[15] Xu Q., Shi J., Yao M., Jiang M., Luo Y. (2016). Effects of heat treatment on the antigenicity of four milk proteins in milk protein concentrates. Food Agric. Immunol..

[16] Ehn B.M., Ekstrand B., Bengtsson U., Ahlstedt S. (2004). Modification of IgE Binding during Heat Processing of the Cow’s Milk Allergen β-Lactoglobulin. J. Agric. Food Chem..

[17] Taheri-Kafrani A., Gaudin J.C., Rabesona H., Nioi C., Agarwal D., Drouet M., Chobert J.M., Bordbar A.K., Haertle T. (2009). Effects of heating and glycation of β-lactoglobulin on its recognition by ige of sera from cow milk allergy patients. J. Agric. Food Chem..

[18] Nowak-Wegrzyn A., Fiocchi A. (2009). Rare, medium, or well done? The effect of heating and food matrix on food protein allergenicity. Curr. Opin. Allergy Clin. Immunol..

[19] Corzo-Martínez M., Soria A.C., Belloque J., Villamiel M., Moreno F.J. (2010). Effect of glycation on the gastrointestinal digestibility and immunoreactivity of bovine β-lactoglobulin. Int. Dairy J..

[20] Xue J., Rai V., Singer D., Chabierski S., Xie J., Reverdatto S., Burz D.S., Schmidt A.M., Hoffmann R., Shekhtman A. (2011). Advanced glycation end product recognition by the receptor for AGEs. Structure.

[21] Heilmann M., Wellner A., Gadermaier G., Ilchmann A., Briza P., Krause M., Nagai R., Burgdorf S., Scheurer S., Vieths S. (2014). Ovalbumin modified with pyrraline, a maillard reaction product, shows enhanced T-cell immunogenicity. J. Biol. Chem..

[22] Zenker H.E., Teodorowicz M., Ewaz A., van Neerven R.J.J., Savelkoul H.F.J., De Jong N.W., Wichers H.J., Hettinga K.A. (2020). Binding of CML-Modified as Well as Heat-Glycated β-lactoglobulin to Receptors for AGEs is Determined by Charge and Hydrophobicity. Int. J. Mol. Sci..

[23] Foerster A., Henle T. (2003). Glycation in food and metabolic transit of dietary AGEs (advanced glycation end-products): Studies on the urinary excretion of pyrraline. Biochem. Soc. Trans..

[24] Roncero-Ramos I., Delgado-Andrade C., Tessier F.J., Niquet-Léridon C., Strauch C., Monnier V.M., Navarro M.P. (2013). Metabolic transit of Nε-carboxymethyl-lysine after consumption of AGEs from bread crust. Food Funct..

[25] Hellwig M., Geissler S., Matthes R., Peto A., Silow C., Brandsch M., Henle T. (2011). Transport of Free and Peptide-Bound Glycated Amino Acids: Synthesis, Transepithelial Flux at Caco-2 Cell Monolayers, and Interaction with Apical Membrane Transport Proteins. ChemBioChem.

[26] O’Hagan D.T., Palin K.J., Davis S.S. (1987). Intestinal absorption of proteins and macromolecules and the immunological response. Crit. Rev. Drug Carr. Syst..

[27] Picariello G., De Cicco M., Nocerino R., Paparo L., Mamone G., Addeo F., Berni Canani R. (2019). Excretion of dietary cow’s milk derived peptides into breast milk. Front. Nutr..

[28] Zhao D., Li L., Le T.T., Larsen L.B., Su G., Liang Y., Li B. (2017). Digestibility of Glyoxal-Glycated β-Casein and β-Lactoglobulin and Distribution of Peptide-Bound Advanced Glycation End Products in Gastrointestinal Digests. J. Agric. Food Chem..

[29] Zenker H.E., Raupbach J., Boeren S., Wichers H.J., Hettinga K.A. (2020). The effect of low vs. high temperature dry heating on solubility and digestibility of cow’s milk protein. Food Hydrocoll..

[30] Ménard O., Bourlieu C., De Oliveira S.C., Dellarosa N., Laghi L., Carrière F., Capozzi F., Dupont D., Deglaire A. (2018). A first step towards a consensus static in vitro model for simulating full-term infant digestion. Food Chem..

[31] Dingess K.A., De Waard M., Boeren S., Vervoort J., Lambers T.T., Van Goudoever J.B., Hettinga K. (2017). Human milk peptides differentiate between the preterm and term infant and across varying lactational stages. Food Funct..

[32] Liu M.Q., Zeng W.F., Fang P., Cao W.Q., Liu C., Yan G.Q., Zhang Y., Peng C., Wu J.Q., Zhang X.J. (2017). PGlyco 2.0 enables precision N-glycoproteomics with comprehensive quality control and one-step mass spectrometry for intact glycopeptide identification. Nat. Commun..

[33] Cox J., Mann M. (2008). MaxQuant enables high peptide identification rates, individualized p.p.b.-range mass accuracies and proteome-wide protein quantification. Nat. Biotechnol..

[34] Boggs I., Hine B., Smolenski G., Hettinga K., Zhang L., Wheeler T.T. (2015). Changes in the repertoire of bovine milk proteins during mammary involution. Eupa Open Proteom..

[35] Matsuo H., Yokooji T., Taogoshi T. (2015). Common food allergens and their IgE-binding epitopes. Allergol. Int..

[36] Knol E.F., de Jong N.W., Ulfman L.H., Tiemessen M.M. (2019). Management of cow’s milk allergy from an immunological perspective: What are the options?. Nutrients.

[37] Milkovska-Stamenova S., Hoffmann R. (2016). Identification and quantification of bovine protein lactosylation sites in different milk products. J. Proteom..

[38] Donato L., Guyomarc’h F. (2009). Formation and properties of the whey protein/κ-casein complexes in heated skim milk—A review. Dairy Sci. Technol..

[39] Alexander L.J., Hayes G., Pearse M.J., Stewart A.F., Willis I.M., Mackinlay A.G. (1989). Complete sequence of the bovine β-lactoglobulin cDNA. Nucleic Acids Res..

[40] Vetri V., Militello V. (2005). Thermal induced conformational changes involved in the aggregation pathways of beta-lactoglobulin. Biophys. Chem..

[41] Van Boekel M.A.J.S. (1999). Heat-induced deamidation, dephosphorylation and breakdown of caseinate. Int. Dairy J..

[42] Wada Y., Lönnerdal B. (2015). Effects of Industrial Heating Processes of Milk-Based Enteral Formulas on Site-Specific Protein Modifications and Their Relationship to in Vitro and in Vivo Protein Digestibility. J. Agric. Food Chem..

[43] Bernard H., Meisel H., Creminon C., Wal J.M. (2000). Post-translational phosphorylation affects the IgE binding capacity of caseins. Febs Lett..

[44] Egger L., Ménard O., Baumann C., Duerr D., Schlegel P., Stoll P., Vergères G., Dupont D., Portmann R. (2019). Digestion of milk proteins: Comparing static and dynamic in vitro digestion systems with in vivo data. Food Res. Int..

[45] Picariello G., Ferranti P., Fierro O., Mamone G., Caira S., Di Luccia A., Monica S., Addeo F. (2010). Peptides surviving the simulated gastrointestinal digestion of milk proteins: Biological and toxicological implications. J. Chromatogr. B: Anal. Technol. Biomed. Life Sci..

[46] Gasparini A., Buhler S., Faccini A., Sforza S., Tedeschi T. (2020). Thermally-induced lactosylation of whey proteins: Identification and synthesis of lactosylated β-lactoglobulin epitope. Molecules.

[47] Ruiter B., Trégoat V., M’Rabet L., Garssen J., Bruijnzeel-Koomen C.A.F.M., Knol E.F., Van Hoffen E. (2006). Characterization of T cell epitopes in αs1-casein in cow’s milk allergic, atopic and non-atopic children. Clin. Exp. Allergy.

[48] Elsayed S., Eriksen J., Øysæd L.K., Idsøe R., Hill D.J. (2004). T cell recognition pattern of bovine milk αS1-casein and its peptides. Mol. Immunol..

[49] Nakajima-Adachi H., Hachimura S., Ise W., Honma K., Nishiwaki S., Hirota M., Shimojo N., Katsuki T., Ametani A., Kohno Y. (1998). Determinant analysis of IgE and IgG4 antibodies and T cells specific for bovine alpha(s)1-casein from the same patients allergic to cow’s milk: Existence of alpha(s)1-casein-specific B cells and T cells characteristic in cow’s-milk allergy. J. Allergy Clin. Immunol..

[50] Gouw J.W., Jo J., Meulenbroek L.A.P.M., Heijjer T.S., Kremer E., Sandalova E., Knulst A.C., Jeurink P.V., Garssen J., Rijnierse A. (2018). Identification of peptides with tolerogenic potential in a hydrolysed whey-based infant formula. Clin. Exp. Allergy.

[51] Ilchmann A., Burgdorf S., Scheurer S., Waibler Z., Nagai R., Wellner A., Yamamoto Y., Yamamoto H., Henle T., Kurts C. (2010). Glycation of a food allergen by the Maillard reaction enhances its T-cell immunogenicity: Role of macrophage scavenger receptor class A type I and II. J. Allergy Clin. Immunol..

[52] Xu Q., Hong H., Wu J., Yan X. (2019). Bioavailability of bioactive peptides derived from food proteins across the intestinal epithelial membrane: A review. Trends Food Sci. Technol..

[53] Reitsma M., Westerhout J., Wichers H.J., Wortelboer H.M., Verhoeckx K.C.M. (2014). Protein transport across the small intestine in food allergy. Mol. Nutr. Food Res..

[54] Moradi S.V., Hussein W.M., Varamini P., Simerska P., Toth I. (2016). Glycosylation, an effective synthetic strategy to improve the bioavailability of therapeutic peptides. Chem. Sci..

[55] Varamini P., Mansfeld F.M., Blanchfield J.T., Wyse B.D., Smith M.T., Toth I. (2012). Synthesis and biological evaluation of an orally active glycosylated endomorphin-1. J. Med. Chem..

[56] Polt R., Porreca F., Szabò L.Z., Bilsky E.J., Davis P., Abbruscato T.J., Davis T.P., Horvath R., Yamamura H.I., Hruby V.J. (1994). Glycopeptide enkephalin analogues produce analgesia in mice: Evidence for penetration of the blood-brain barrier. Proc. Natl. Acad. Sci. USA.

[57] Zenker H.E., Ewaz A., Deng Y., Savelkoul H.F.J., Van Neerven R.J.J., De Jong N., Wichers H.J., Hettinga K.A., Teodorowicz M. (2019). Differential effects of dry vs. Wet heating of β-lactoglobulin on formation of sRAGE binding ligands and sIgE epitope recognition. Nutrients.

[58] Pinto M.S., Léonil J., Henry G., Cauty C., Carvalho A.F., Bouhallab S. (2014). Heating and glycation of β-lactoglobulin and β-casein: Aggregation and in vitro digestion. Food Res. Int..

