# Supplementary material for: Peptide Release after Simulated Infant In Vitro Digestion of Dry Heated Cow’s Milk Protein and Transport of Potentially Immunoreactive Peptides across the Caco-2 Cell Monolayer
Source: Nutrients. 2020 Aug 18;12(8):2483. doi: 10.3390/nu12082483 (PMC7468992; doi:10.3390/nu12082483)
Supplement: Supplementary file 1 [file nutrients-12-02483-s001.zip › supplementaryMaterial-20200621.docx]

**Supplementary Materials:**

| (**a**)  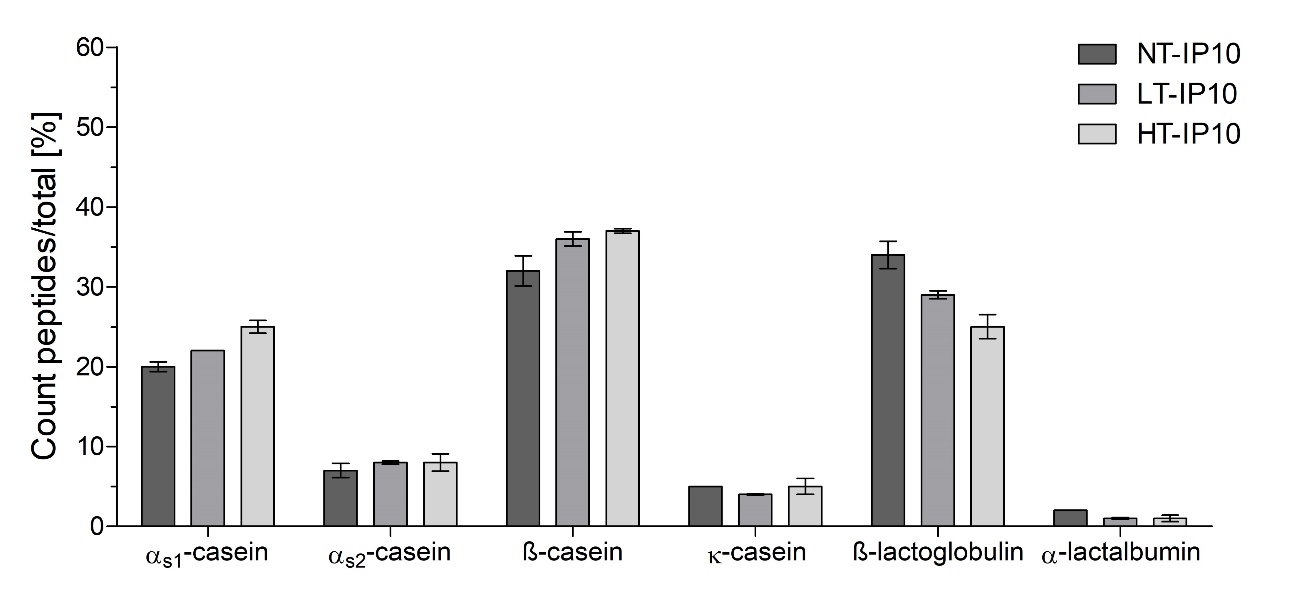 |
| --- |
| (**b**)  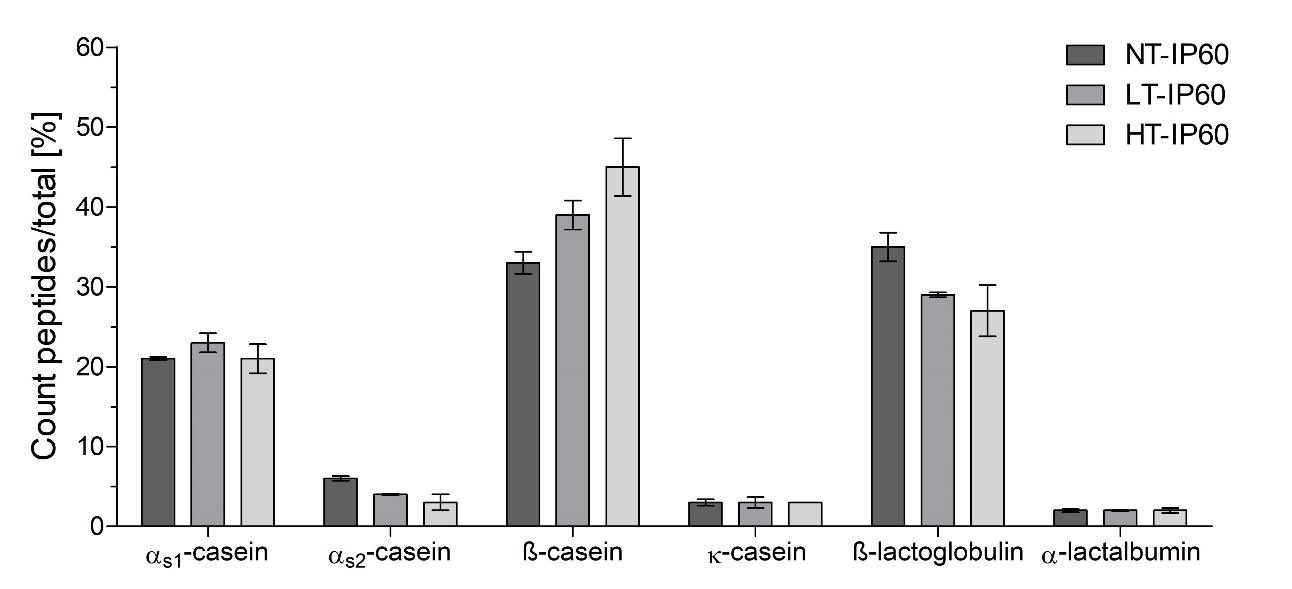 |

**Figure S1:** Peptides derived from each of the five major milk proteins after infant *in vitro* digestion of cow’s milk protein. Samples were non-treated (NT), heated at low temperature (LT), and heated at high temperature (HT), with data shown as percentage peptide count to total within the same sample after (a) 10 minutes in the intestinal phase (IP) and (b) after 60 minutes in the IP. Total number of peptides after 10 minute in the IP was 436 ± 4, 369 ± 26, and 315 ± 36 for NT, LT, and HT, respectively. Total number of peptides after 60 minute in the IP was 255 ± 15, 246 ± 7, and 207 ± 1 for NT, LT, and HT, respectively. Data are shown in mean ± standard deviation from duplicate digestion experiments.

| (**a**)  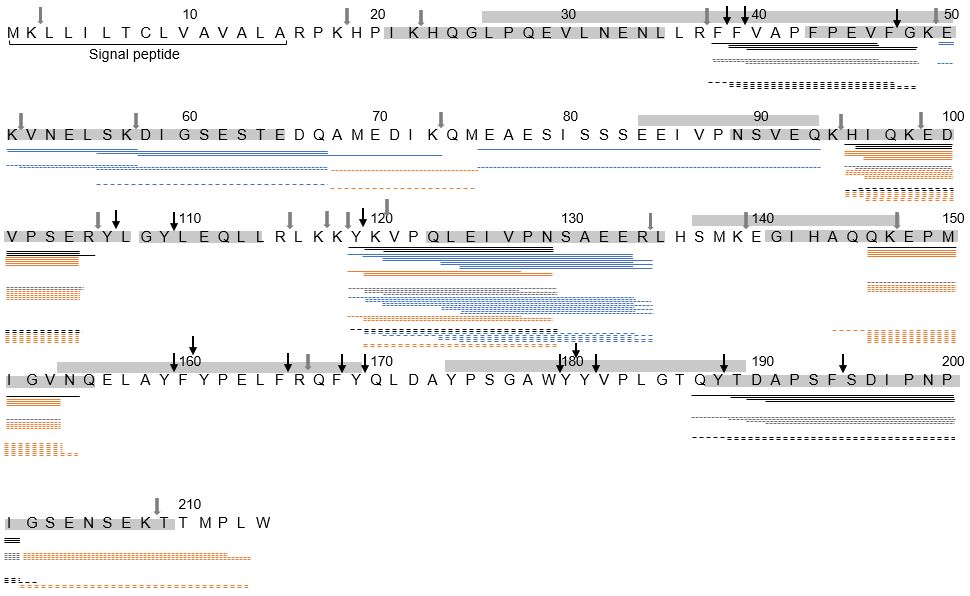 |
| --- |
| (**b**)  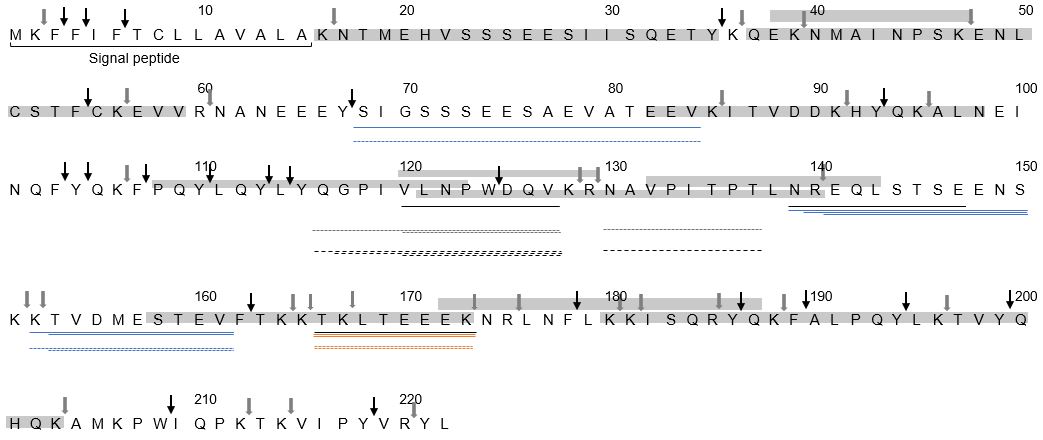 |
| (**c**)  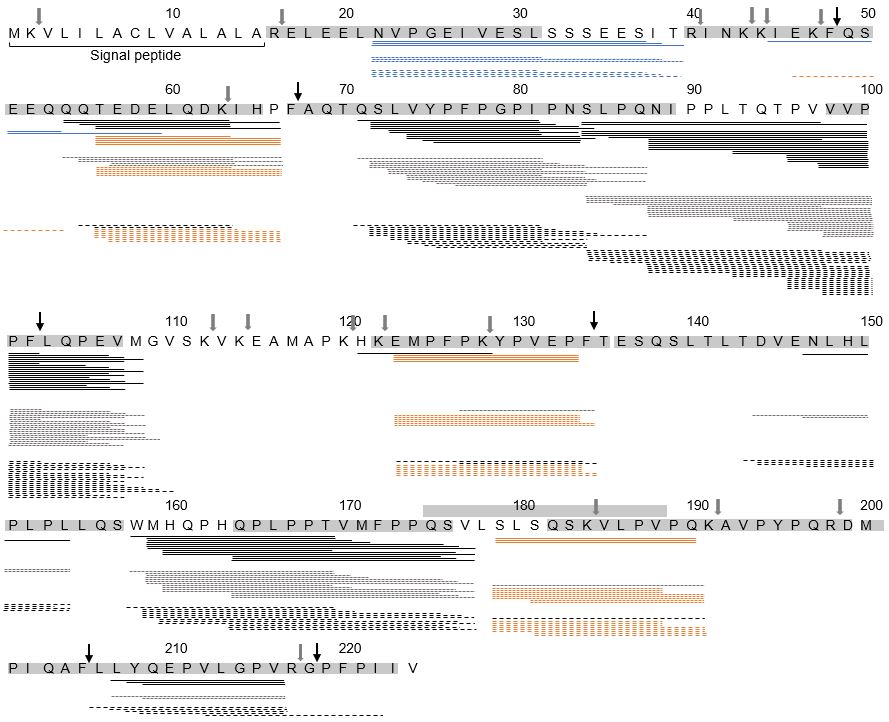 |
| (d)  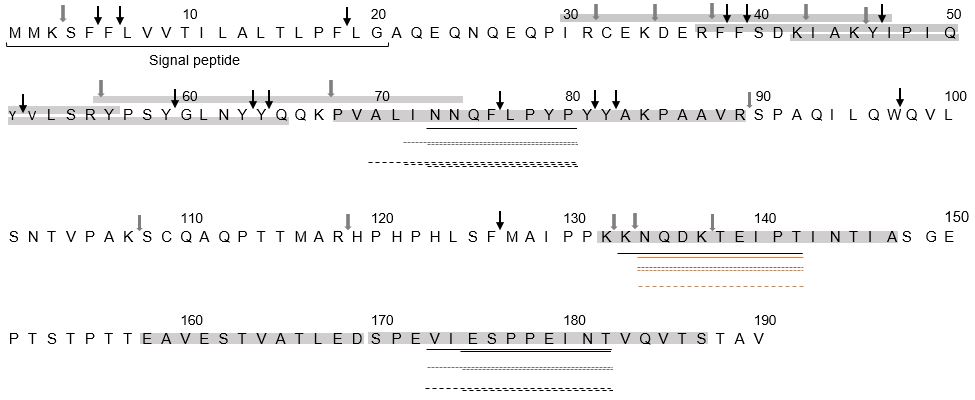 |
| (e)  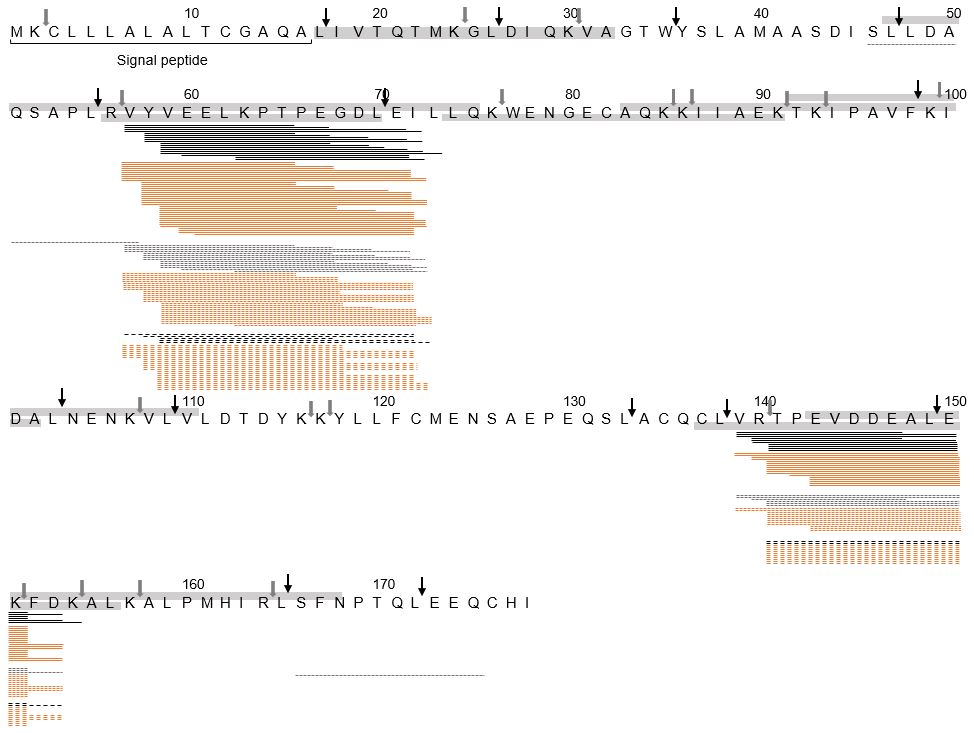 |
| (f)  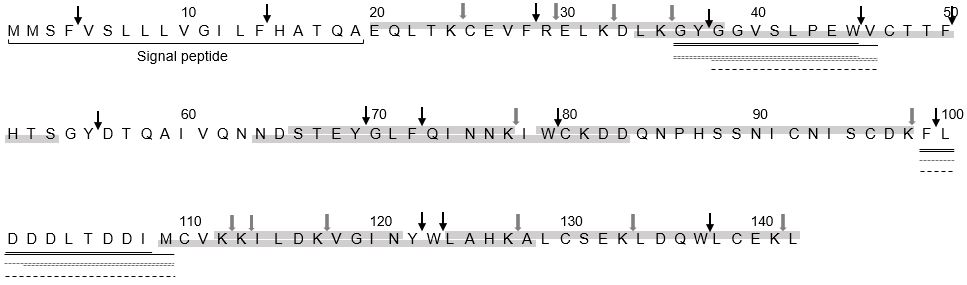 |

**Figure S2:** Sequence alignment of digestive peptides after 60 minutes in the intestinal phase. Peptides derived from (a) α_s1_-casein, (b) α_s2_-casein, (c) β-casein, (d) κ-casein,(e) β-lactoglobulin, (f) α-lactalbumin identified after simulate infant *in vitro* digestion of non-treated cow’s milk protein (full line), heated at low temperature (dotted line), and heated at high temperature (dashed line). Glycated peptides (orange), phosphorylated peptides (blue), trypsin cleavage sites (thick grey down arrow), chymotrypsin cleavage sites (thin black down arrow). Trypsin and chymotrypsin cleavage sites were determined using Expasy Bioinformatics Resource Portal (https://web.expasy.org/peptide_cutter/ last visited 08.06.2020).

**Table S1.** sIgE binding epitopes^1^ identified in digestive peptides derived from cow’s milk protein after 60 minutes in the intestinal phase. Samples were non-treated (NT), heated at low temperature (LT), and heated at high temperature (HT) in the presence of lactose. Peptides derived from casein (cn), β-lactoglobulin (lg), and α-lactalbumin (lac). sIgE binding epitopes of the Peptides matching exactly the sIgE binding epitope sequence are indicated (*). Amino acids (AAs) position indicates the position within the proteins including the signal peptide. Peptides carrying a post translational modifications (PTM) are marked with phosphorylation (Phos), where phosphorylated serine (S) and threonine (T) as we as lysine residues (K) modified to glucosyllysine (Gluc), lactosyllysine (Lac), pyrraline (Pyr), and N^ɛ^-carboxymethyllysine are highlighted in bold and underline. Localisation probabilities are given in brackets if multiple options were identified.

| **Protein** | **Sample** | **Peptide sequence** | **AAs position** | **sIgE epitope AAs position** | **PTM** |  | |
| --- | --- | --- | --- | --- | --- | --- | --- |
| α_s1_-cn | LT | VNEL**S(1)**KDIG**S(1)**E**S(0.87)T(0.13)**EDQ | 52-67 | 54-63 | Phos |  | |
|  | NT,LT,HT | KVPQLEIVPN**S**AEE | 120-133 | 124-135 | Phos |  | |
|  | LT | KVPQLEIVPN**S**AEER | 120-134 | 124-135 | Phos |  | |
|  | NT,LT,HT | VPQLEIVPN**S**AEER | 121-134 | 124-135 | Phos |  | |
|  | NT,LT,HT | LEIVPN**S**AEE | 124-133 | 124-135 | Phos |  | |
|  | NT,LT,HT | LEIVPN**S**AEER | 124-134 | 124-135 | Phos |  | |
|  | NT,LT,HT | EIVPN**S**AEER | 125-134 | 124-135 | Phos |  | |
|  | NT,LT,HT | QYTDAPSFSDIPNPI | 187-201 | 186-200 | N/A. |  | |
| β-cn | LT | LVYPFPGPIPNSLPQ | 73-87 | 70-85 | N/A |  | |
|  | NT,LT,HT | PVVVPPFLQPE | 96-106 | 98-107 | N/A |  | |
|  | NT,LT,HT | PVVVPPFLQPEV | 96-107 | 98-107 | N/A |  | |
|  | LT,HT | PVVVPPFLQPEVMG | 96-109 | 98-107 | N/A |  | |
|  | HT | PVVVPPFLQPEVMGV | 96-110 | 98-107 | N/A |  | |
|  | NT,LT,HT | VVPPFLQPE | 98-106 | 98-107 | N/A |  | |
|  | NT,LT,HT | VVPPFLQPEV | 98-107 | 98-107 | N/A | * | |
|  | NT,LT,HT | EMPFP**K**YPVEP | 123-133 | 122-135 | Lac |  | |
|  | NT,LT,HT | EMPFP**K**YPVEP | 123-133 | 122-135 | Gluc |  | |
|  | NT,LT,HT | EMPFP**K**YPVEP | 123-133 | 122-135 | CML |  | |
|  | NT,LT,HT | EMPFP**K**YPVEP | 123-133 | 122-135 | Pyr |  | |
|  | LT,HT | EMPFP**K**YPVEPF | 123-134 | 122-135 | Lac |  | |
|  | LT,HT | EMPFP**K**YPVEPF | 123-134 | 122-135 | Gluc |  | |
|  | NT,LT,HT | QPLPPTVMFPPQS | 164-176 | 164-179 | N/A |  | |
| β-lg | NT,LT,HT | VYVEELKPTPEGDLE | 57-71 |  | N/A |  | |
|  | NT,LT,HT | VYVEEL**K**PTPEGDLE | 57-71 |  | Lac |  | |
|  | NT,LT,HT | VYVEEL**K**PTPEGDLE | 57-71 |  | Gluc |  | |
|  | NT,LT,HT | VYVEEL**K**PTPEGDLE | 57-71 |  | CML |  | |
|  | NT,LT,HT | VYVEEL**K**PTPEGDLE | 57-71 |  | Pyr |  | |
|  | NT | VYVEELKPTPEGDLEI | 57-72 |  | N/A. |  |  |
|  | NT | VYVEEL**K**PTPEGDLEI | 57-72 |  | Lac |  |  |
|  | NT | VYVEEL**K**PTPEGDLEI | 57-72 |  | Gluc |  |  |
|  | NT | YVEEL**K**PTPEGDL | 58-70 |  | Lac |  |  |
|  | NT,LT,HT | YVEELKPTPEGDLE | 58-71 |  | N/A |  |  |
|  | NT,LT,HT | YVEEL**K**PTPEGDLE | 58-71 |  | Lac |  |  |
|  | NT,LT,HT | YVEEL**K**PTPEGDLE | 58-71 |  | Gluc |  |  |
|  | NT,LT,HT | YVEEL**K**PTPEGDLE | 58-71 |  | CML |  |  |
|  | NT,LT,HT | YVEEL**K**PTPEGDLE | 58-71 |  | Pyr |  |  |
|  | NT,LT | YVEELKPTPEGDLEI | 58-72 |  | N/A |  |  |
|  | NT | YVEEL**K**PTPEGDLEI | 58-72 |  | Lac |  |  |
|  | NT | YVEEL**K**PTPEGDLEI | 58-72 |  | Gluc |  |  |
|  | NT | YVEEL**K**PTPEGDLEI | 58-72 |  | CML |  |  |

^1^ Peptides were reported as IgE binding epitopes if their sequence contained at least 80% of the sequence of an IgE binding epitope.

**Table S2:** Potential T-cell epitopes identified after 60 minutes in the intestinal phase. Peptides were identified as potential T-cell epitopes using IEDB MHC Class II Binding Prediction software (<http://tools.iedb.org/mhcii/>). Digestive peptides identified from cow’s milk protein, non-treated (NT), dry heated at low temperature (LT), and dry heated at high temperature (HT) applied to simulated infant *in vitro* digestion, derived from casein (cn), β-lactoglobulin (lg), and α-lactalbumin (lac). Amino acids (AAs) position indicating the position within the proteins including the signal peptide. Unmodified peptides and peptides with post translational modifications (PTM), via phosphorylation (Phos), as well as modification to glucosyl-lysine (Gluc), lactosyl-lysine (Lac), carboxymethyl lysin (CML), and pyrraline (Pyr) were reported.

| **Protein** | **Sample** | **Sequence** | **HLA-allele** | **AAs position** | **PTM** | **Perc. rank** |
| --- | --- | --- | --- | --- | --- | --- |
| α_s1_-cn | NT, LT | EAESI**SSS**EEIVPNSVEQ | HLA-DQA1*03:01/ DQB1*03:02;  HLA-DQA1*04:01/ DQB1*04:02 | 76-93 | Phos | 2.5  5.3 |
|  | NT, LT | YKVPQLEIVPN**S**AEE | HLA-DRB1*04:05;  HLA-DQA1*04:01/ DQB1*04:02;  HLA-DQA1*03:01/ DQB1*03:02 | 119-133 | Phos | 1.9  4.1  5.8 |
|  | LT | KVPQLEIVPN**S**AEER | HLA-DRB1*04:05;  HLA-DQA1*04:01/ DQB1*04:02;  HLA-DQA1*03:01/ DQB1*03:02 | 120-134 | Phos | 1.9  5.6  5.9 |
| α_s2_-cn | NT, LT | SIGSSSEESAEVATEEV | HLA-DQA1*04:01/ DQB1*04:02;  HLA-DQA1*03:01/ DQB1*03:02 | 68-84 | Phos | 0.14  0.18 |
| β-cn | NT,HT | NVPGEIVESLSSSEES | HLA-DQA1*04:01/ DQB1*04:02 | 22-37 | Phos | 5.43 |
|  |  | NVPGEIVESLSSSEESI | HLA-DRB1*04:05 | 22-38 | Phos | 5.54 |

| 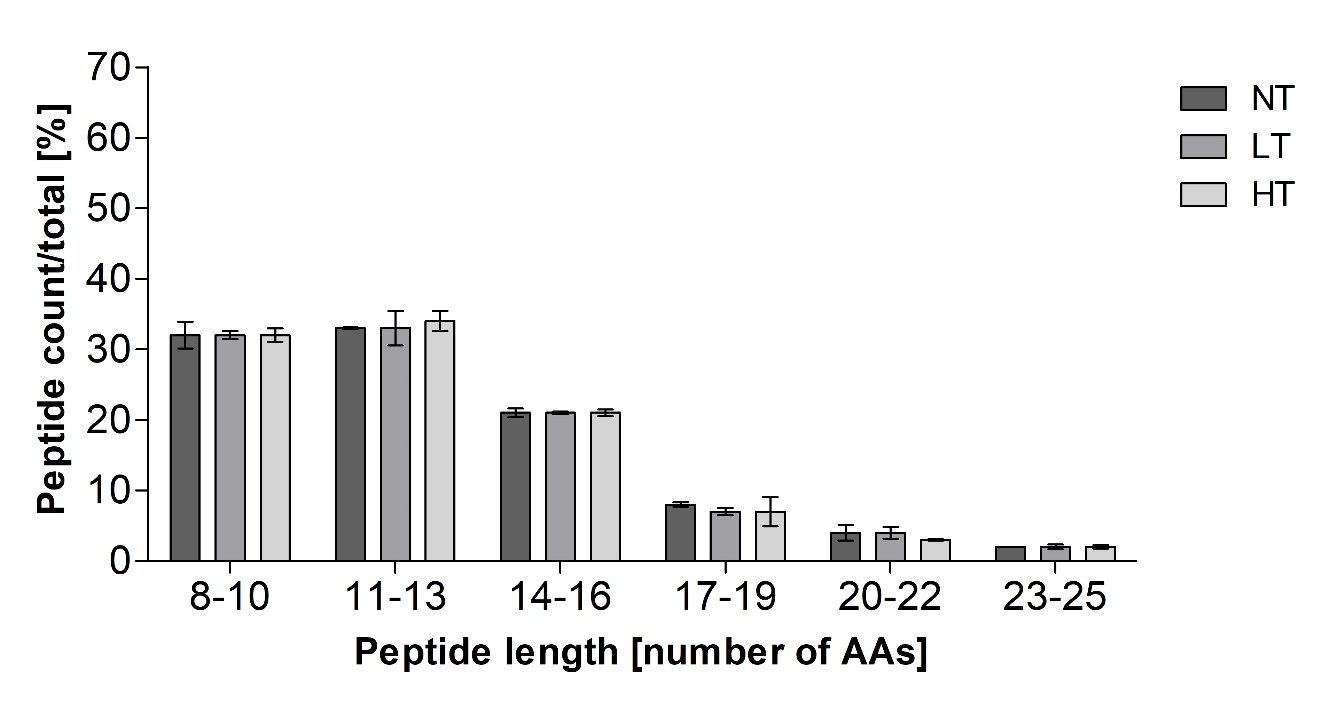 |
| --- |

**Figure S3:** Peptide length distribution identified in the *in vitro* digests of cow’s milk protein after 10 minutes in the intestinal phase. Samples were non-treated (NT), heated at low temperature (LT) as well as at high temperature (HT) in the presence of lactose and applied to simulated infant in vitro digestion. Data are, expressed as percentage to total number of peptides in the same sample (NT: 436 ± 4, LT: 369 ± 26, HT: 315 ± 36).

| 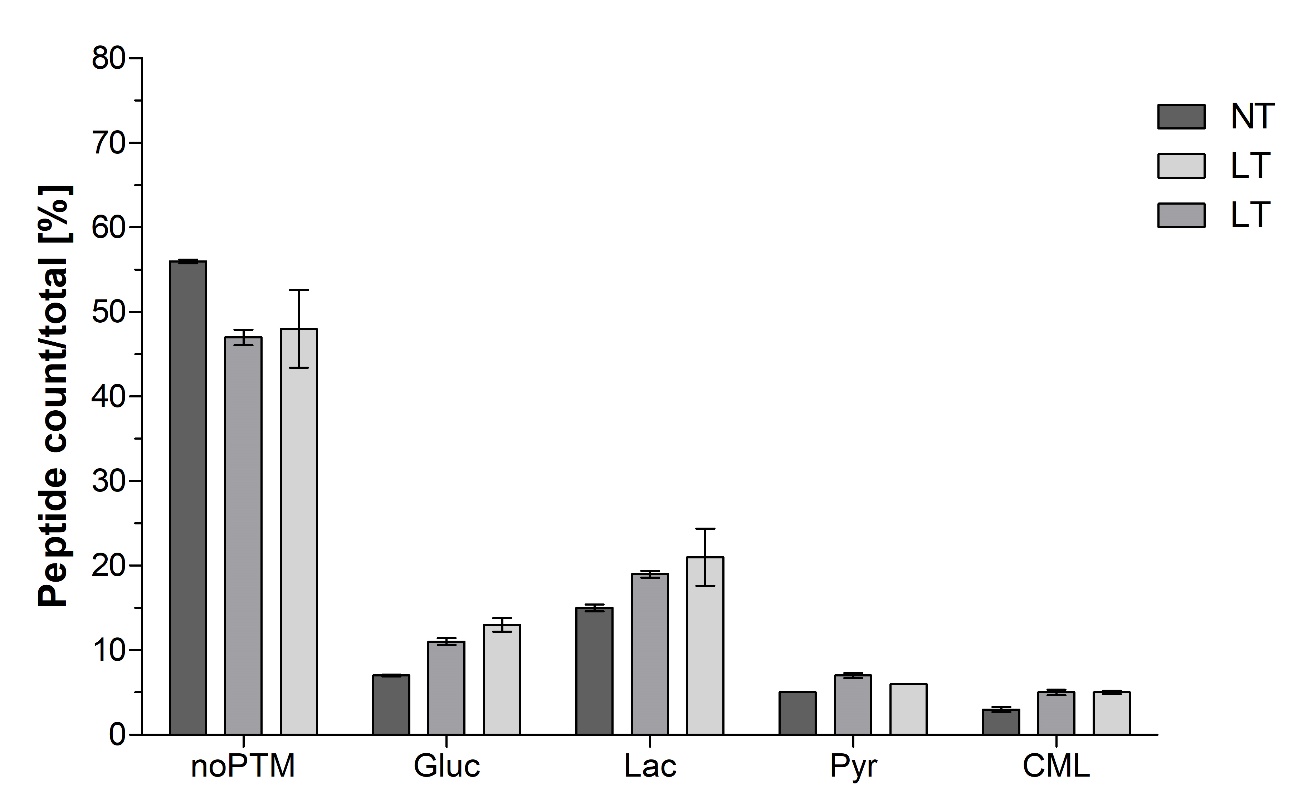 |
| --- |

**Figure S4:** Non-modified vs. glycated digestive peptides identified after 10 minutes in the intestinal phase. Comparison of digestive peptides without posttranslational modification (noPTM), as well as modified to glucosyllysine (Gluc), lactosyllysine (Lac), pyrraline (Pyr), and N^ɛ^-carboxymethyllysine (CML) that were identified in the *in vitro* digests of cow’s milk protein, non-treated (NT), heated in the presence of lactose at low temperature (LT) and high temperature (HT), expressed as peptide count relative to the total number of peptides in the samples (NT: 436 ± 4, LT: 369 ± 26, HT: 315 ± 36). Error bars represent standard deviation of duplicate digestions.

| (**a**)  |
| --- |
| (**b**)  |
| (c)  |
| (d)  |

**Figure S5:** Summed intensities of peptides associated with a specific modification, within the same amino acid sequence. Modification to glucosyllysine (Gluc), lactosyllysine (Lac), N^ɛ^-carboxymethyllysine (CML), and pyrraline (Pyr) found in peptides generated after simulated infant *in vitro* digestion of cow’s milk protein, non-treated (NT), dry heated at low temperature (LT), and dry heated at high temperature (HT), sampled after 10 minutes in the intestinal phase (IP10) and after 60 minutes in the intestinal phase (IP60) are shown. Duplicate digestion experiments (DG1 and DG2) for the same sample are shown, where green indicates the lowest intensity (0.0 for LL, GL, CML, and PY) and red indicates highest intensity (Gluc:1.3E+9, Lac: 7.5E+9, CML: 5.1E+7, Pyr: 2.7E+8).
